# A transient α-helix in the N-terminal RNA recognition motif of polypyrimidine tract binding protein senses RNA secondary structure

**DOI:** 10.1093/nar/gkaa155

**Published:** 2020-03-14

**Authors:** Christophe Maris, Sandrine Jayne, Fred F Damberger, Irene Beusch, Georg Dorn, Sapna Ravindranathan, Frédéric H-T Allain

**Affiliations:** 1 Department of Biology, ETH Zurich, 8093 Zürich, Switzerland; 2 Central NMR facility, National Chemical Laboratory, Pune 411008 India

## Abstract

The polypyrimidine tract binding protein (PTB) is a multi-domain protein involved in alternative splicing, mRNA localization, stabilization, polyadenylation and translation initiation from internal ribosome entry sites (IRES). In this latter process, PTB promotes viral translation by interacting extensively with complex structured regions in the 5′-untranslated regions of viral RNAs at pyrimidine-rich targets located in single strand and hairpin regions. To better understand how PTB recognizes structured elements in RNA targets, we solved the solution structure of the N-terminal RNA recognition motif (RRM) in complex with an RNA hairpin embedding the loop sequence UCUUU, which is frequently found in IRESs of the picornovirus family. Surprisingly, a new three-turn α3 helix C-terminal to the RRM, folds upon binding the RNA hairpin. Although α3 does not mediate any contacts to the RNA, it acts as a sensor of RNA secondary structure, suggesting a role for RRM1 in detecting pyrimidine tracts in the context of structured RNA. Moreover, the degree of helix formation depends on the RNA loop sequence. Finally, we show that the α3 helix region, which is highly conserved in vertebrates, is crucial for PTB function in enhancing Encephalomyocarditis virus IRES activity.

## INTRODUCTION

RNA binding proteins (RBPs) are essential in the regulation of diverse processes in RNA biology, such as mRNA splicing, RNA transport, storage, degradation, post-transcriptional modification and translation. Critical in all of these functions, is the ability of RBPs to recognize binding sites on the RNA in the proper structural context, i.e. RNA secondary structure and spacing between binding sites. It is important to understand how this contextual information is used by RBPs to determine the recognition of the binding site and to modulate RBP functions. Whereas many RBPs have been identified, the structural features of the RNA that determine where they bind are only beginning to be understood, although this is essential for elucidating their function (1).

Polypyrimidine tract binding protein (PTB or PTBP or PTBP1) also called heterogeneous nuclear ribonucleoprotein I (hnRNP I) is a nucleocytoplasmic protein, which regulates diverse processes in mRNA metabolism (2–4). In alternative splicing, PTB acts primarily as a repressive splicing regulator. However, it can also enhance exon inclusion and the role it plays depends on the relative position of its binding site, exons and the polyadenylation signal (5–8). PTB can also increase mRNA stability: for example, binding of PTB to a pyrimidine-rich sequence located in the 3′ untranslated region of insulin mRNA increases its life time (9). In the process of cap-independent translation initiation, PTB is a *trans*-acting factor of several cellular and viral internal ribosome entry sites (IRESs) located in the 5′ untranslated region of the mRNA (2). PTB interacts in particular with a number of viral IRES RNAs from the *picornoviridae* family, which comprises poliovirus (PV), human rhinovirus (HRV), hepatitis A virus (HAV), foot and mouth disease virus (FMDV), Theiler's murine encephalomyelitis virus (TMEV) and encephalomyocarditis virus (EMCV). These IRES RNAs adopt highly complex structures, which contain short and long pyrimidine stretches identified as PTB binding sites. It has been proposed that PTB plays the role of an RNA chaperone and that it may stabilize or rearrange IRES RNA structure in order to enable, with the help of eukaryotic initiation factors, the recruitment of the ribosome (10). It has been characterized mainly as an enhancer of viral IRES-mediated translation, and as a promoter of RNA replication (11,12).

**Figure 1.**
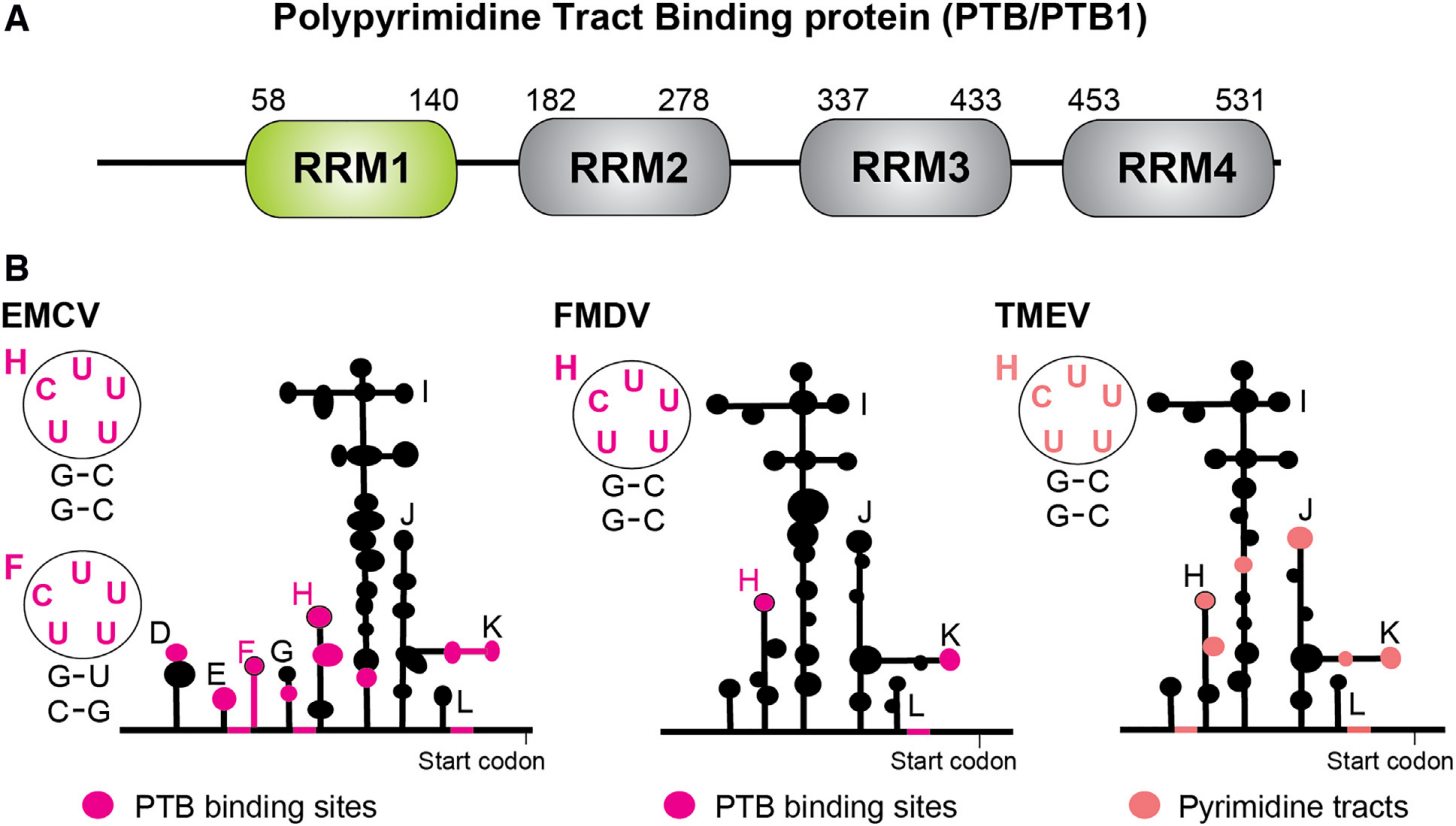
(**A**) Domain structure of PTB with its four RRMs. (**B**) Schematic representation of the secondary structures of the picornavirus IRESs of type II: encephalomyocarditis virus (EMCV), foot and mouse disease virus (FMDV) and Theiler's murine encephalomyelitis virus (TMEV). The RNA binding sites of PTB identified in EMCV and FMDV, and the pyrimidine tracts in TMEV, are colored magenta and pink respectively. The secondary structures of several hairpin binding sites containing a UCUUU motif are highlighted.

PTB, which is 531 amino acid long, is a monomer in solution and adopts a linear arrangement (13–15). It consists of a nuclear localization signal (NLS), a nuclear export signal (NES) both located at the N terminus and four RNA recognition motifs (RRM) (Figure 1A) (16). The RRM is the most common RNA-binding domain in RNA-binding proteins, and consists of a four-stranded β-sheet backed by two α-helices. β-strands 1 and 3 of the RRM usually contain the RNA-binding motifs, RNP2 and RNP1 respectively which frequently include aromatic residues to stabilize interactions with the RNA bases via stacking (17). The first two N-terminal RRMs of PTB are separated by a 42 amino acid linker and tumble independently, whereas the two C-terminal RRMs (RRM3 and RRM4) interact extensively with each other (18). By binding two pyrimidine tracts distant in sequence, RRM3 and RRM4 can remodel RNA tertiary structure. This interdomain interaction was shown to be important for the ability of PTB to efficiently repress alternative splicing (19). PTB also acts in concert with its isoforms and homologues, which have distinct activities in alternative splicing and IRES mediated translation (20–23). In addition to the ubiquitously expressed PTB variants there are two tissue specific homologues that share ∼70–80% amino acid sequence identity with PTB: nPTB, mainly expressed in neurons (neural PTB also called brPTB/PTBP2), and regulator of differentiation 1 (ROD1 also called PTBP3) expressed in hematopoietic cells (24).

Because of its RNA binding properties and domain topology, PTB can accomplish its functions by competing directly with other factors at specific RNA binding sites and/or by inducing structural rearrangements of the targeted RNA. The solution structure of each RRM in complex with a short CU repeat RNA revealed that each RRM bound canonically to the single-stranded RNA on their β-sheet surface (15,17,25).

In the context of natural RNA substrates, pyrimidine tracts can be embedded in internal loops, bulges or hairpin loops and this local structural context may influence PTB binding or conformation. The secondary structures of a small number of IRESs, which bind PTB, have been modeled and characterized *in vitro* (26–29). EMCV IRES shows pyrimidine tracts mostly embedded in hairpin loops and internal loops rather than in extended single stranded regions and two studies identified PTB binding sites in CU-rich hairpin loops (30,31) (Figure 1B). The mapping of PTB binding sites on EMCV IRES identified by hydroxyl-radical probing has demonstrated that the IRES sequence binds two PTB molecules with a unique orientation (32). Hydroxyl-radical footprinting also revealed PTB binding sites on PV-1 (poliovirus type 1) IRES and showed that RRM1 and RRM2 both bind on a long stem in domain V containing several pyrimidine-rich internal loops (33). Moreover, Clerte *et al.* observed in their biochemical study that RRM1 and RRM2 of PTB preferentially bind short CU tracts embedded in stem–loops whereas the C-terminal RRM3 and RRM4 prefer to bind long flexible CU-rich sequences (34). Both RRM1 and RRM2 have also been shown to recognize a pyrimidine-rich internal loop present in the U1 snRNA (SL4), and it was proposed that PTB may repress splicing through the binding of one of these RRMs to SL4, thus preventing the assembly of a productive spliceosomal complex (30).

It is critical to understand how PTB can discriminate between structured RNA targets such as hairpin-loops or internal loops, and single stranded RNA containing pyrimidine-tracts, since this is likely to be key in PTB recognition of the complex RNA topologies of its natural substrates, and its ability to act as a scaffolding protein for multiprotein complexes involved in RNA-based gene regulation. Whereas RRM2 was recently shown to display only local adaptions of the backbone upon binding to a stem–loop (35) and the tandem RRM3/RRM4 prefers single stranded RNA sites (34), the behavior of RRM1 upon interacting with a structured RNA target is unknown. To gain better insight on the ability of PTB to recognize structured RNA targets, we therefore determined the structure of the N-terminal RRM of PTB (RRM1) in complex with an RNA hairpin containing a UCUUU apical loop (Figure 1B). Surprisingly, the structure revealed a new C-terminal helix in RRM1, which folds upon binding the RNA hairpin. NMR analysis presented here shows that the C-terminal helix acts as a sensor of the RNA secondary structure, and mobility shift assays and in vivo IRES translation assays indicate that this helix is crucial to promote EMCV IRES activity by PTB.

## MATERIALS AND METHODS

### Protein and RNA preparation

PTB RRM1 and its variants were expressed recombinantly as an intein-tagged fusion protein, which contains only the native amino acid sequence after purification. The N-terminal amino acid was chosen for optimal self-cleavage of the intein tag. The PCR-amplified cDNA fragment, which encodes residues 41–163 of human polypyrimidine-tract binding protein, (PTB1, accession number X62006) was cloned into the pTYB11 vector between the NdeI and Xho1 sites. Mutants were generated by site-directed mutagenesis using specific primers and verified by Sanger sequencing. The protein was purified as previously described (30). All RNAs were in vitro transcribed from chemically synthesized oligonucleotides using a Pro266Leu mutant version of RNA T7 polymerase (36). Magnesium concentration was optimized for *in vitro* transcription reactions with both commercially available unlabeled NTPs and ^13^C,^15^N-labeled NTPs produced in house. The RNAs were purified by anion exchange chromatography in denaturing conditions as previously described (30). In addition to the *in vitro* transcribed RNAs, we chemically synthesized RNAs with ^13^C-labeled sugar moieties at either five or three positions in the apical region to make RNA stem–loops labeled with either of two patterns: **G_9_**U_10_**C_11_**U_12_**U_13_**U_14_**C_15_** or G_9_**U_10_**C_11_**U_12_**U_13_**U_14_**C_15_, where bold characters indicate positions of nucleotides with ^13^C isotope labeled riboses (37). To reduce the probability of forming RNA-duplexes, RNA samples were diluted in about 25 ml of water, heated to 95°C and snap cooled in liquid nitrogen before lyophilization and resuspension in NMR-buffer.

### NMR measurements

All NMR measurements were performed in low salt buffer (20 mM NaCl and 10 mM NaH_2_PO_4_ adjusted to pH 6.5 with NaOH solution with protein and RNA concentrations of 1 mM) at a temperature of 40°C except for experiments used to detect NOEs involving imino proton resonances of the SL UCUUU RNA which were obtained at 5°C in order to reduce their exchange with water. Chemical shifts were referenced using internal DSS. Data were acquired on Bruker AVIII-500 MHz, 600 MHz, 700 MHz, 750 MHz and AVANCE 900 MHz spectrometers all equipped with cryo-probes with the exception of the 750 MHz spectrometer. Data were processed using Topspin 2.1 (Bruker) and analyzed with Sparky (http://www.cgl.ucsf.edu/home/sparky/) (T.D. Goddard, D.G. Kneller, SPARKY 3, University of California, San Francisco) and CARA (www.nmr.ch) (38). The backbone and side-chain resonances of the wild type (WT) PTB RRM1 construct were assigned using 3D HNCA, 3D HNCOCA, 3D HNCACB, 3D CBCACONH, 3D HNCO and 3D HC(C)H-TOCSY and 3D (H)CCH-TOCSY (23 ms mixing time), 3D NOESY ^1^H–^15^N HSQC and 3D NOESY ^1^H–^13^C aliphatic and aromatic HSQC (150 ms mixing time) (39). The backbone of the L151G mutant of PTB RRM1 was assigned similarly. Backbone resonance assignments of PTB RRM1 bound to SL RNAs with variant loop sequences were obtained by transferring the assignments from the SL UCUUU complex augmented by systematic comparison among the complexes with variant SL RNAs. A similar strategy was used to assign PTB RRM1 mutants bound to SL UCUUU. Aromatic proton resonances were assigned using a 2D ^1^H–^1^H TOCSY and the 3D NOESY ^1^H–^13^C aromatic HSQC. Nonexchangeable proton resonances of the SL UCUUU RNA in both free and protein-bound states were assigned using 2D ^1^H–^1^H TOCSY, 2D ^1^H–^1^H NOESY (mixing time of 200 ms), 3D NOESY ^1^H–^13^C HSQC (mixing time of 200 ms) and 3D HCP NMR experiments with samples in NMR buffer dissolved in 99.97% D_2_O solution. The use of chemically synthesized RNAs, with specifically ^13^C-labeled sugars at different nucleotide positions in the UCUUU loop region, facilitated the resonance assignment especially in the protein-bound form. Imino resonances were assigned by sequential walk of imino–imino NOEs in the stem of the RNA, supplemented by the through-bond 2D H(NC4C5)H5 experiments in the loop (40). Intermolecular NOEs were extracted from a 3D ^13^C f1-filtered, f3-edited NOESY aliphatic ^13^C–^1^H HSQC, and a 2D ^1^H–^1^H NOESY with ^13^C filter in the direct dimension. Intermolecular NOEs between the protein and the imino proton of U12 uracil were assigned using a 2D ^1^H–^1^H NOESY and a 3D NOESY ^13^C aliphatic HSQC with 3–9–19 WATERGATE solvent suppression at 5°C in 90% H_2_O/10% D_2_O. We determined the residual dipolar couplings of the protein H–N amides, RNA H–N iminos and proton-carbon correlations (H2C2, H8C8, H6C6, H5C5, H1′C1′) using IPAP-TROSY experiments implemented in an interleaved manner. Residual dipolar couplings were calculated from the difference between the one bond scalar couplings (^1^J_CH_) obtained in the absence and in the presence of alignment medium (13 mg/ml Pf1 phage from ASLA biotech). Chemical shift mapping of PTB RRM1 upon RNA binding was calculated according to the formula Δδ = ((ΔδN/6.51)^2^ + ΔδH^2^))^1/2^ where ΔδN and ΔδH are the nitrogen and proton chemical shift differences respectively, between the free and RNA bound states. Secondary ^13^C shifts were calculated by subtracting sequence-corrected random coil chemical shifts at the appropriate temperature and pH (41,42). Amide shifts of the L151G mutant of PTB RRM1 at 45°C and some shifts of WT at 40°C were calculated by extrapolation of data at a series of temperatures.

### Structure calculation and refinement

We calculated an ensemble of structures of the complex in a semi-automated manner using the ATNOSCANDID (43,44) and CYANA (45) software packages. Initial calculations with ATNOSCANDID were performed with the complete resonance assignments of the protein together with the following NOESY spectra as input; a 3D NOESY ^15^N-edited HSQC obtained in 90% H_2_O/10% D_2_O, 3D NOESY ^13^C-edited aliphatic HSQC spectra acquired in both 90% H_2_O/10% D_2_O and in 99.97% D_2_O, and a 3D NOESY ^13^C-aromatic edited HSQC in 99.97% D_2_O. ATNOSCANDID peak lists were then used to derive distance constraints with CYANA. In addition, hydrogen bond restraints were defined between carbonyls and amides, which had slow hydrogen–deuterium exchange (visible 1 h after dissolution of a lyophilized sample in D_2_O). Dihedral angle constraints for the protein were derived using TALOS (46). Intermolecular and intra-RNA restraints for the UCUUU loop of the bound RNA were derived from NOEs calibrated in a similar manner to the CYANA software using fixed distances from the covalent structure. In addition, the final CYANA calculation included RNA stem restraints derived from a 3D NOESY ^13^C-edited HSQC of the free RNA for those nucleotides with shifts which were unchanged in the complex because this avoided overlap in NOESY spectra of the complex. δ torsion angle restraints for the RNA were determined experimentally from a 2D ^1^H–^1^H TOCSY experiment (80°–90° for C3′ endo, 55°–115° for loose C3′ endo, 130°–190° for C2′ endo and 55°–190° for nucleotides exchanging between both conformations). In addition, Watson–Crick hydrogen bond restraints and loose dihedral angle restraints were defined for phosphodiester linkages of the RNA stem region (47). From the 250 calculated structures, the fifty structures with the lowest target function were refined in AMBER 12 (48) and the 20 lowest energy structures retained. During the final minimization, the force constants of the square-well penalty functions defined for NOE, torsion angle and RDC restraints were held fixed to 50 kcal mol^−1^ Å^−2^, 1000 kcal mol^−1^ rad^−2^ and 0.1 kcal mol^−1^ Hz^−2^ respectively. We implemented an annealing protocol similar to one which was previously described (49). The quality of the structure ensemble was analyzed with the PROCHECK software (50). The agreement of RDCs with the imposed RDC constraints were checked by the *Q*-factor (51). All the structures were displayed and analysed with MOLMOL software (52).

### Isothermal titration calorimetry

Isothermal titration calorimetry (ITC) experiments were performed at 30°C on a VP-ITC instrument (Microcal). Both protein and RNA samples were dialyzed together in the same NMR buffer. The concentration of the protein in the cell was 10 μM and the RNA concentrations in the syringe varied from 70 to 120 μM (280 μl). The titrations were performed with 30 or 50 injections. Data were analyzed with Origin 7.0 software.

### Plasmids, cell culture, transient transfection and reporter gene assay

Dicistronic pRF (Renilla luciferase and Firefly luciferase) and pRemcvF plasmids were a generous gift from Prof. Dr A. Willis (MRC Toxicology Unit, Leicester, UK) (53). Mammalian expression vector pcFlag-PTB was a generous gift from Prof. Dr D. Black (UCLA, USA). pRemcvF, pUC-emcv-5′ (281–450 spanning stem–loops D to H), pcFlag-PTB and pET28a-PTB mutants were created by site directed mutagenesis using specific primers and verified by sequencing. For the loop mutant constructs, the loop E UUGUCUAU sequence as well as loop F and loop H UCUUU sequences were substituted by the UUCG tetraloop. Sequences of the mutagenic primers are available upon request.

To test EMCV IRES activity, *in vivo* translation assays were performed using human HEK293T cells. Cells were maintained in Dulbecco's modified Eagle's medium supplemented with 10% heat-inactivated fetal calf serum (GibcoBRL) and antibiotics. Cells were plated on six-well plates the day before transfection and were transfected in duplicate with calcium phosphate precipitation using 250 ng of dicistronic emcv wild type or loop mutant reporter plasmids according to the original manufacturer protocol. All EMCV-IRES activity tests were performed in biological duplicates. For RNA interference experiments, cells were plated on 24-well plates the day before transfection. On Day 1, cells were transfected with 30 nM siRNA targeting PTB1 (P1), nPTB (N1) and control siRNA (C2) (9,54) using Lipofectamine 2000 following the supplier's instructions. On Day 2, cells were transfected with siRNA, dicistronic reporter assay and wild type or mutant PTB1 expression vector. For all experiments, cells were lysed in 1× Passive Lysis Buffer (Promega) 24 h after the last transfection. The activities of Firefly and *Renilla* luciferases were measured using the dual-luciferase reporter assay system (Promega) according to the manufacturer's instructions. Light emission was measured with a Berthold MicrolumatPlus luminometer.

### Western blot analysis

Forty μg of cell extracts, prepared either in 1× Passive Lysis Buffer (Promega) or in Lysis buffer (50 mM Tris pH 8, 125 mM NaCl, 0.5% (v/v) NP-40, 20% (v/v) glycerol, 0.5 mM dithiothreitol (DTT), 1 mM phenylmethylsulfonylfluoride and protease inhibitor (Roche)), were separated with 10% SDS-polyacrylamide gel electrophoresis. PTB1 and nPTB knockdown and ectopic PTB1 expression was analyzed by western blot with anti PTB-NT (1:3000), anti nPTB-1S2 (1:500) and anti GAPDH (1:5000) antibodies (Sigma) using the Immun-Star WesternC kit from Bio-Rad. Chemiluminescence was detected on a Typhoon Trio (Amersham Biosciences).

### Expression of PTB1 mutants, *in vitro* transcription and electromobility shift assays

PTB1 mutants were overexpressed in BL21 DE3 *Escherichia coli* and purified on a Nickel affinity column. Purified proteins were dialyzed against a buffer containing 20 mM NaH_2_PO_4_ (pH 7.2), 100 mM NaCl, 5 mM MgCl_2_, 10% (v/v) glycerol, 1 mM DTT. Concentrations of proteins were determined using optical density absorbance at 280 nm. Both wild type and loop mutants of EMCV 5′ RNA, were transcribed *in vitro* from linearized pUC-emcv-5′ vector and purified using the Total RNA isolation kit from Macherey-Nagel. RNAs were subsequently dephosphorylated using Calf Intestine Phosphatase (Fermentas) and 5′ labeled using γ-^32^P-ATP. Unincorporated nucleotides were removed using a IllustraMicrospin G-25 column (GE Healthcare). RNAs were incubated with a range of protein concentrations in a buffer containing 20 mM NaH_2_PO_4_ (pH 7.2), 100 mM NaCl, 5 mM MgCl_2_, 5% (v/v) glycerol, 1 mM DTT, 50 μg/ml yeast tRNA (R9001, Sigma), 40 μg/ml BSA (New England Biolabs) and SUPERase·In RNase Inhibitor (AM2696, Ambion) for 20 min at room temperature. After addition of loading buffer, samples were loaded on a 4% acrylamide/bisacrylamide (29:1) gel in 0.5% TBE and electrophoresed for 1.5 h at 200 V at room temperature. Gels were dried and exposed on a PhosphoScreen (GE Healthcare).

## RESULTS

### Major amide chemical shift changes at the C-terminus of PTB RRM1 upon RNA hairpin binding

Sequence alignments of PTB, nPTB and ROD1 homologues, and of PTB in many vertebrate species show residue conservation, which extends both N-terminally and C-terminally well beyond the canonical RRM, suggesting a functional role for these flanking regions (Supplementary Figure S1). We therefore cloned, expressed and purified isotope labeled protein encompassing the N-terminal RRM flanked by 20 and 30 residues, respectively (residues 41–163, referred to as PTB RRM1) as described in Methods.

**Figure 2.**
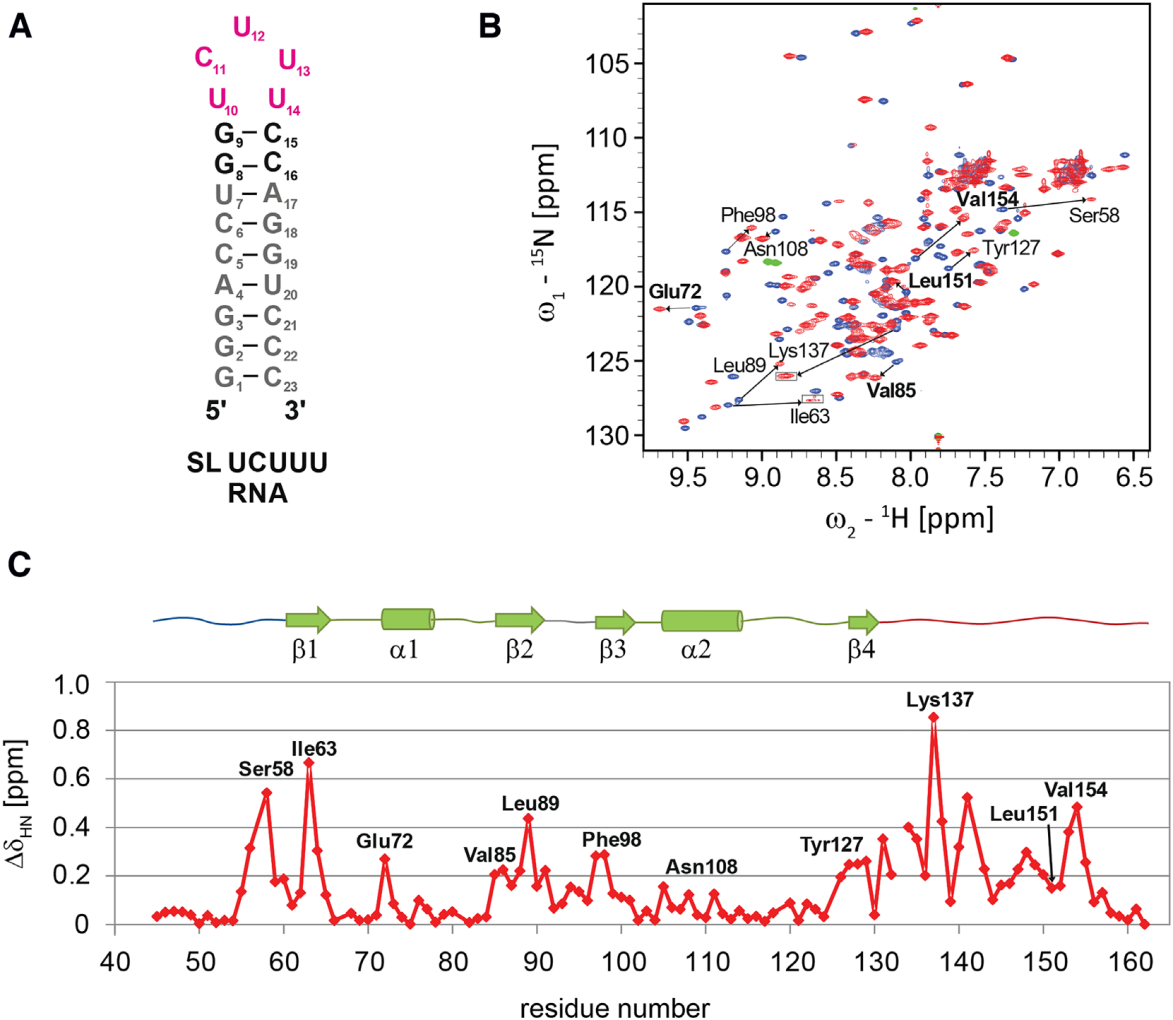
(**A**) Secondary structure of SL UCUUU. (**B**) Superposition of ^1^H-^15^N HSQC of PTB RRM1 in SL UCUUU RNA free and bound states acquired at 40°C (blue and red respectively). Boxed regions have lower contour thresholds. Selected amide shift changes from residues at the RRM-α3 interface are indicated in bold. (**C**) Amide chemical shift perturbation mapping of PTB RRM1 upon binding SL UCUUU.

We investigated by NMR titration the binding of PTB RRM1 to a 23 nucleotide RNA (Figure 2A) that embeds the UCUUU apical loop sequence found in SL F and SL H of EMCV IRES, and also in stem–loops of other picornavirus IRES (Figure 1B). In what follows, we refer to this generic RNA as SL UCUUU. SL UCUUU formed a single hairpin conformation in solution under our NMR conditions with standard Watson-Crick base-pairing indicated for the stem by an imino walk in 2D NOESY spectra and an imino–imino NOE detected between the uridines at the 5′ and 3′ end of the loop. The pattern of pyrimidine aromatic TOCSY correlations was preserved at elevated temperatures indicating the same conformation was present at 40°C where we obtained optimal NMR spectral quality for protein and RNA. Amide resonances of the protein (and its variants) showing large chemical shift changes between free and bound state (indicated in Figure 2B) exhibited slow exchange behavior during the titration of SL UCUUU, as well as all other stem–loops described in this study, with separate signals observed for free and bound forms, whereas signals exhibiting smaller shift changes exhibited either exchange broadening or fast exchange behavior. We observed major chemical shift differences for most of the amide resonances of the protein (Figure 2B). As expected, large perturbations in the RNA-binding β-sheet region were observed but more surprisingly, additional large chemical shift changes were seen in the N-terminal region immediately upstream of the RRM domain (56–60) and in the C-terminal extension (131–154) (Figure 2C). In addition, the amide resonance of Glu72 at the N-terminus of α1 helix, which is outside of the expected RNA binding surface, also showed a fairly large chemical shift change.

To determine PTB RRM1 affinity for SL UCUUU, we measured the dissociation constant of the complex by ITC. Fitting of the experimental data with a single binding site model resulted in a stoichiometry close to 0.5 indicating that one RNA molecule binds two protein molecules. We therefore fit the data with a model for two independent binding sites. This gave better fits to the experimental data and we could extract two dissociation constants (*K*_d1_ = 0.10 ± 0.02 μM and *K*_d2_ = 3.0 ± 0.9 μM; Supplementary Figure S2). Even though our ITC analysis showed two binding events, only one set of protein amide resonances is observed for the bound state under our NMR conditions, which represent the high affinity binding complex (Figure 2B, Supplementary Figure S3). With the exception of the first two N-terminal residues Gly41, Asn42 as well as His133, all the observed amide resonances could be assigned in the 1:1 complex. These NMR data reveal the presence of only one detectable bimolecular complex at equimolar ratio and millimolar concentrations and are consistent with the measured dissociation constants, which predict a negligible amount of protein populating the 30-fold weaker RNA binding state.

### The structure of PTB RRM1 in complex with SL UCUUU reveals a newly folded C-terminal helix

**Figure 3.**
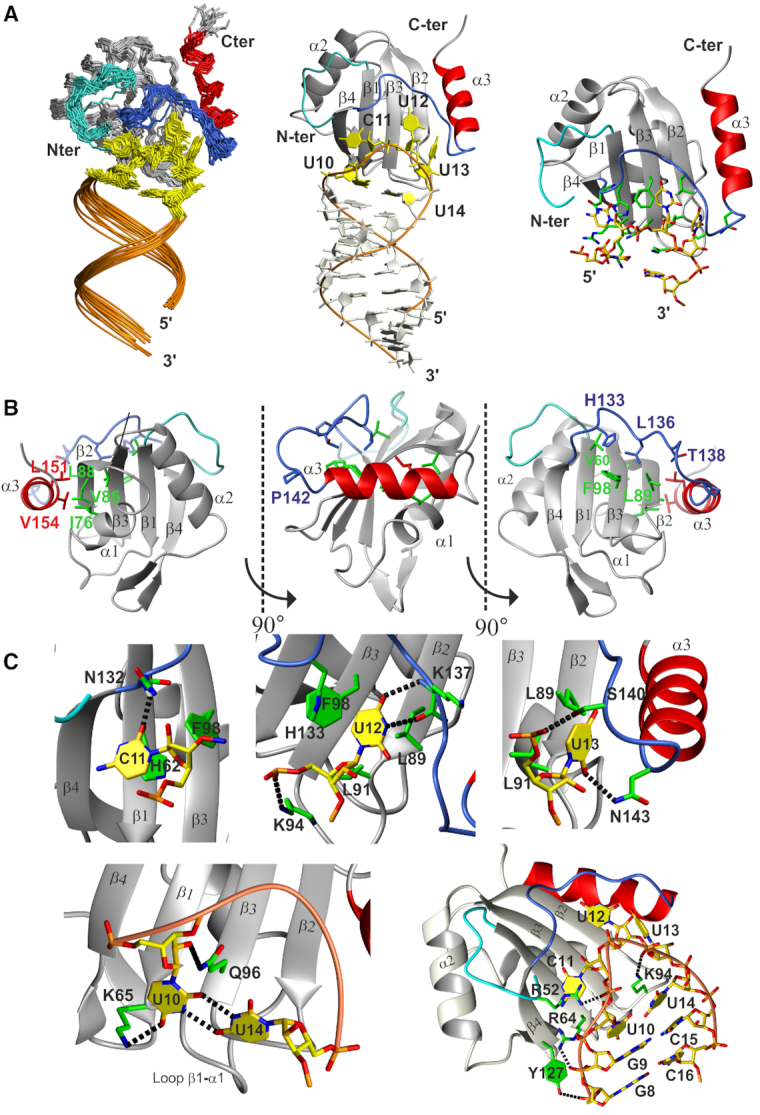
(**A**) Overview of the structure of PTB RRM1 in complex with SL UCUUU RNA. Left: Ensemble of the 20 structures superimposed on the backbone heavy atoms of the well-defined residues of the protein and RNA (residues 58-155 of the protein and 1–23 of the RNA). The N-terminal extension, extended β4-α3 loop, α3 helix and pentaloop nucleotides are highlighted in cyan, blue, red and yellow respectively. Middle: lowest energy structure of the RNA-protein complex. Right: RNA–protein interface, key protein sidechains in green. (**B**) Contacts between RRM domain and structured parts of the N- and C-termini. Side chains from the RRM domain, N-terminus, extended β4–α3 loop and α3 helix are represented in cyan, green, blue and red respectively. (**C**) Details of protein interactions with the loop nucleotides, mismatched U10–U14 base pair and RNA backbone. Hydrogen bonds are represented by black dashed lines.

We determined the solution structure of PTB RRM1 in complex with SL UCUUU using a combination of 2318 distance constraints derived from NOEs and 71 orientational constraints derived from residual dipolar couplings (RDCs). The protein/RNA interface was defined by 98 intermolecular NOEs (Supplementary Table S1). Samples utilizing synthesized RNA with ^13^C labeled sugars at specific positions in the apical loop facilitated unambiguous assignments of the ribose resonances, and were very useful for obtaining intra and intermolecular restraints (Supplementary Figure S4). The U12 and U13 nucleotides displayed a dense network of intermolecular restraints whereas fewer such restraints could be defined for C11 because of severe line broadening in this region of the interface (Supplementary Figure S5). The structural ensemble of conformers is well defined with a heavy atom RMSD of 1.22 ± 0.44 Å for ordered regions (Supplementary Table S1 and Figure 3A). In the ensemble, contacts between protein and RNA involve mainly the β-sheet of the protein, and the UCUUU pentaloop of the RNA. The nucleotides of the CUU triplet interact with the β-sheet surface whereas U10 and U14 form a mismatched base-pair which is also present in the free RNA as indicated by the small chemical shift differences of the H5–H6 TOCSY correlations observed for these nucleotides between the free and protein-bound forms (Supplementary Figure S5C). The UU base pair is recognized by loops of the RRM at the base of the β-sheet. The regions immediately N- and C-terminal to the RRM are structured and interact with the RNA, a feature which has been observed often in RRM–RNA interactions (55). Particularly, the extended loop following β4, which forms an arch above the CUU triplet on the β-sheet, engages in many contacts with the RNA. Residues His133, Leu136 and Thr138 interact with Val60, Leu89 and Phe98 from the β-sheet (Figure 3B). More surprisingly, the C-terminal region located ten residues downstream of β4 (140–155), which was shown to undergo large amide chemical shift changes upon RNA binding, embeds an additional twelve residue α-helix (α3) (residues Gln144–Val154). This helix is in a region that forms part of the 42-residue linker between RRM1 and RRM2 of PTB. Importantly, the helix itself does not interact with the RNA. The newly formed helix α3 packs against the RRM, contacting β2 and the helix α1 (Figure 3A and B). A set of five hydrophobic residues, Leu151 and Val154 from α3, Ile76 from α1, and Val85 and Leu88 from the β2-strand, form the interface between the domain and α3 (Figure 3B). Immediately N-terminal to α3, a *trans* proline (Pro142) induces a hinge directing α3 to run anti-parallel to the β2-strand.

### SL UCUUU sequence- and shape-specific recognition by PTB RRM1

At the binding interface, the nucleotides C11–U12–U13 lie on the β-sheet surface and the flanking uracils U10 and U14 form a mismatched UU base pair (Figure 3A). The sugar puckers of the CUU triplet and mismatched UU base pair adopt a C2′ endo conformation, and an intermediate conformation between C2′ and C3′ endo conformations, respectively. The C11 nucleotide stacks on His62 from β1 (RNP2 motif, Figure 3C). The amide of Asn132 in the β4-α3 loop is hydrogen bonded to the carbonyl oxygen (O4) of C11. The U12 base stacks with Leu89 from β2 and its Watson-Crick face makes two hydrogen bonds to the Lys137 main chain. The U12 imino was observed at 5°C and gave rise to numerous NOEs to the side chain resonances of Leu136, Lys137 and Thr138 (Supplementary Figure S4D). The large chemical shift change of the Lys137 amide upon RNA binding (Figure 2C) and the detection of the U12 imino proton at 10.84 ppm support the presence of these hydrogen bonds. U13 interacts with Leu89 and Leu91 of β2, which forms one edge of the β-sheet. The side chains of Asn143 and Ser140 in the β4–α3 loop contact the O2 base carbonyl and the phosphate of U13, respectively. Finally, the mismatched base pair between U10 and U14 is recognized by the side chains of Lys65 and Gln96, which contact the O4 carbonyl and the O2′ sugar of the U10 nucleotide, respectively. In addition, Lys94 from loop β2–β3 protrudes into the pentaloop where its side chain stacks on the U10–U14 base pair and is positioned to make a favorable ionic interaction with the U13 phosphate. The side chains of Arg64 and Tyr127 interact non-specifically with the phosphate oxygens of G9, and G8 respectively. The region N-terminal to the RRM folds back onto the β-sheet surface facilitating interactions of Arg52 with the C11 phosphate oxygen. The large chemical shift change of the Ser58 amide proton is consistent with the structural rearrangement of this region upon RNA binding (Figure 2C). In summary, the structure shows that the CUU triplet of the apical loop lies on the β-sheet surface and makes base-specific interactions with the extended β4–α3 loop. Loop β1–α1 also contributes to base-specific interactions by contacting U10, which is base-paired with U14. The β2–β3 loop, β1, β4 and Ser140 located N-terminal to α3, interact with phosphate oxygens from the RNA loop and from G8 and G9 of the two GC base pairs closing the loop. These interactions to the phosphate backbone contribute to nonspecific RNA binding affinity and help RRM1 to recognize the shape of the stem–loop structure.

### Affinity measurement of mutant complexes by isothermal titration calorimetry

**Figure 4.**
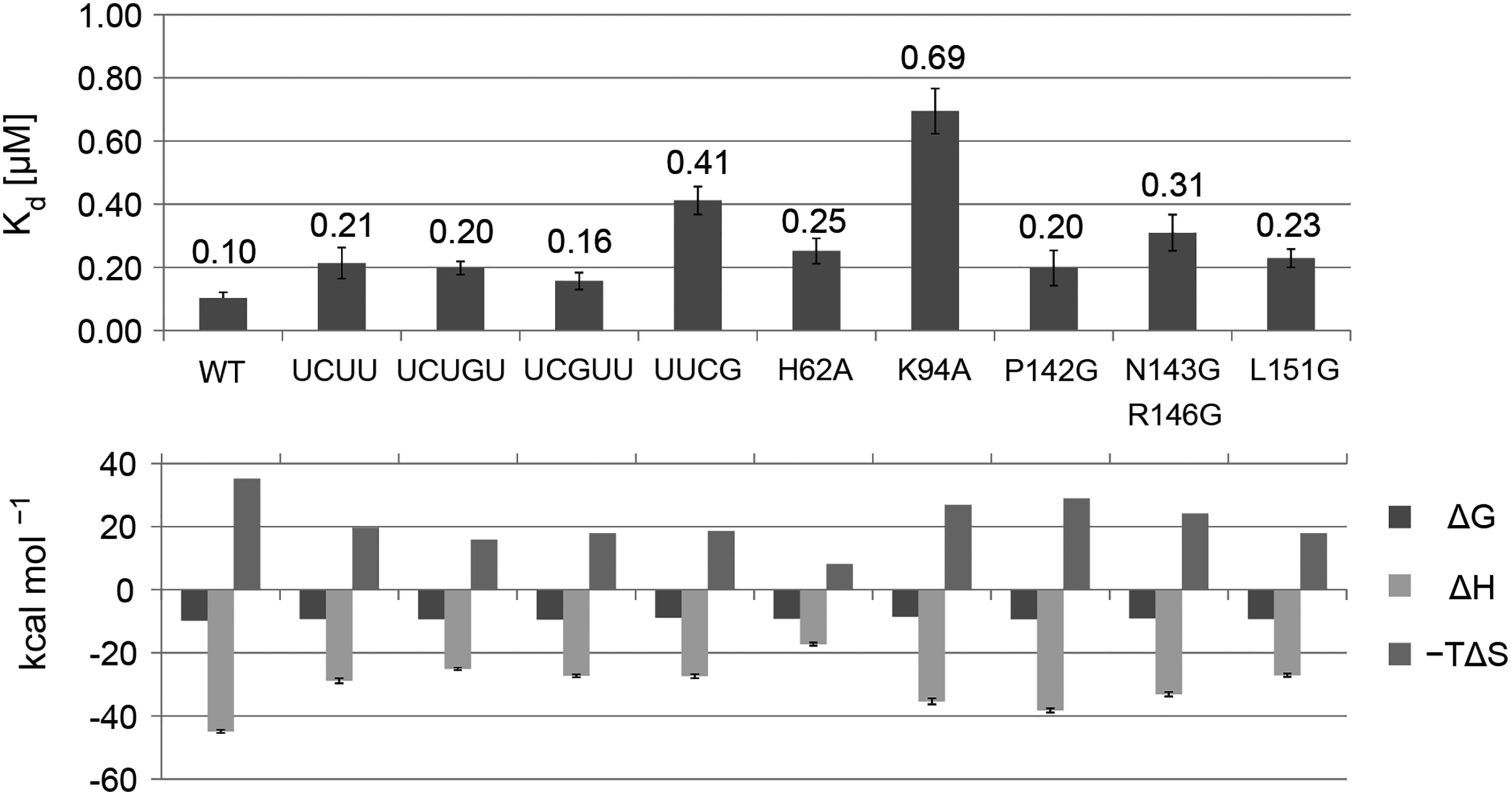
Dissociation constants *K*_d1_ and thermodynamic parameters (Δ*H* enthalpy, Δ*S* entropy and Δ*G* free energy) of the wild type and mutant complexes obtained with ITC.

In order to evaluate the importance of protein/RNA interactions and the α3 helix for complex formation, we measured the dissociation constants and thermodynamic parameters of complexes formed between RNA and protein by ITC, where either the RNA or the protein were modified by site-directed mutagenesis (Figure 4 and Supplementary Figure S6A). All ITC curves were fitted assuming two independent binding sites, as for the wild type complex. In all tested mutations, the dissociation constant *K*_d2_ corresponding to the weaker binding affinity has a magnitude similar to the wild type (average of 4.1 ± 0.9 μM) (Supplementary Figure S6A). The first dissociation constant (*K*_d1_) of the different RNA mutants for WT protein showed only small increases compared to the SL UCUUU (2- to 4-fold, Figure 4). Surprisingly, deletion of one nucleotide in the apical loop, substitution of either U12 or U13 by a G, or replacement of the entire UCUUU loop by the stable UUCG tetraloop all had minimal impact on the affinity, although replacement of the UCUUU loop by UUCG was shown to be detrimental to function (see below). On the protein side, we generated five mutants, which were designed, on the basis of the structure of the complex, to affect the RNA binding affinity (H62A and K94A from the RNP2 motif and loop β2–β3, respectively) or the folding of helix α3 (P142G, N143G/R146G and L151G from the C-terminal extension). The mutation K94A caused the strongest effect with almost a 7-fold loss in affinity, consistent with the loss of stacking interactions with the mismatched U10–U14 base pair and ionic interactions with the RNA backbone (Figure 3C).

Mutations in the α3 helix region showed smaller effects, with up to a 3-fold loss of affinity for the N143G/R146G double-mutant. Examination of the thermodynamic parameters revealed more significant differences between the various complexes. Large favorable enthalpy and unfavorable entropy characterize the thermodynamic signature of the wild type complex. The thermodynamic parameters of all the mutant complexes indicate smaller enthalpic gain as expected, but these gains were compensated by smaller entropic losses, resulting in only moderate impacts on free energy of binding (Figure 4). The H62A mutation in the RNP2 motif showed the strongest change of the thermodynamic signature. A plot of −*T*Δ*S* versus Δ*H* shows an excellent linear correlation (Supplementary Figure S6B) indicating that in these different complexes the entropy and the enthalpy compensate each other resulting in almost the same binding free energy for each complex. Among the mutations expected to affect the folding of the α3 helix, P142G and N143G/R146G show only an intermediate effect on the enthalpy-entropy parameters whereas the L151G mutant displays the most dramatic effect consistent with the elimination of a large hydrophobic sidechain which made important contacts to residues in the RRM (Figure 3B).

### The C-terminal helix is already partially formed in the free RRM1 and binding occurs by conformational selection

**Figure 5.**
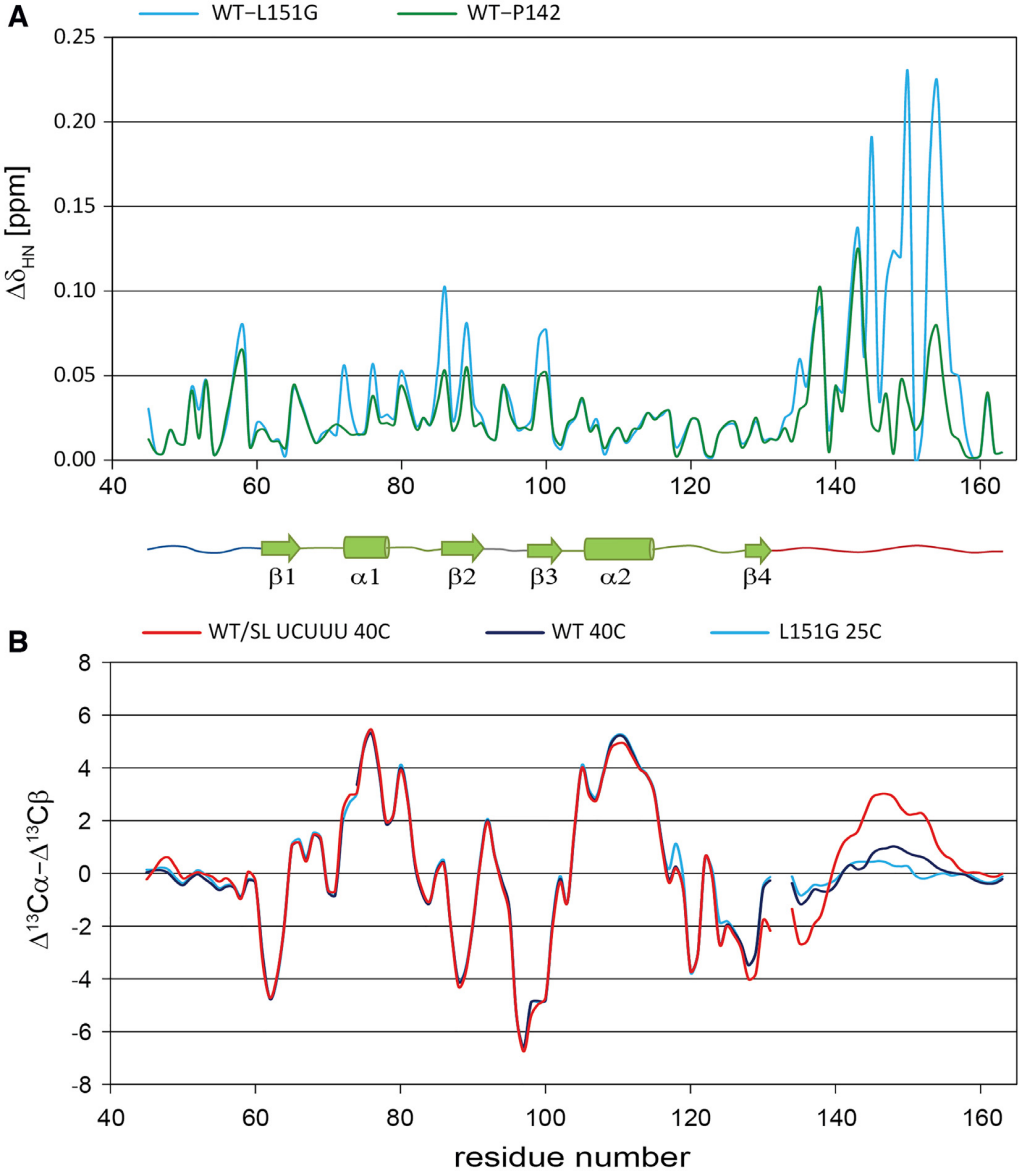
(**A**) Combined amide chemical shift difference between the WT PTB RRM1 protein and the P142G (green) and L151G (blue) mutant proteins both in their RNA free state. (**B**) Smoothed secondary ^13^C chemical shift values (Δ^13^Cα–Δ^13^Cβ) of the WT PTB RRM1 in free state (purple), in complex with SL UCUUU (red) and for the free state of the L151G mutant (blue). The experiments were acquired at 40°C for the WT protein in the free and SL UCUUU bound states, and at 25°C for the L151G mutant.

Comparison of the ^1^H–^15^N HSQC spectrum of the wild type with that of the L151G mutant, designed to negatively impact the formation of the α3 helix (Supplementary Figure S7), revealed significant changes in the position of amides in the α3 helix region (up to 0.22 ppm), β2 strand and α1 helix (up to 0.1 ppm) (Figure 5A, blue line). For the P142G mutant (Figure 5A, green line), the perturbations compared to WT follow a similar pattern but with a smaller amplitude.

We therefore wondered if these differences might originate from the presence of a low degree of helicity in the C-terminal region of the free protein. We assessed helical content using the deviation of ^13^Cα and ^13^Cβ chemical shifts from random coil values (Δ^13^Cα and Δ^13^Cβ, or carbon secondary shifts) which are sensitive to the backbone conformation. Positive values of Δ^13^Cα−Δ^13^Cβ indicate helical character, negative values extended β-strand conformation and values near zero are indicative of random coil (56,57). The C-terminal region from residue 144 to 149 of free PTB RRM1 shows values ranging up to 1 ppm, suggesting a moderate degree of helicity in this region. In contrast, the same region has values consistent with random coil conformation in the L151G mutant (Figure 5B). As expected from the structure of the complex of PTB bound to SL UCUUU, the values of Δ^13^Cα−Δ^13^Cβ in this region are larger, with a maximum value of 2.9 ppm, independently confirming that α3 is folded. However, the values are significantly lower than for the α1 and α2 helices suggesting that even in the complex with SL UCUUU, α3 is less well folded than α1 and α2.

**Figure 6.**
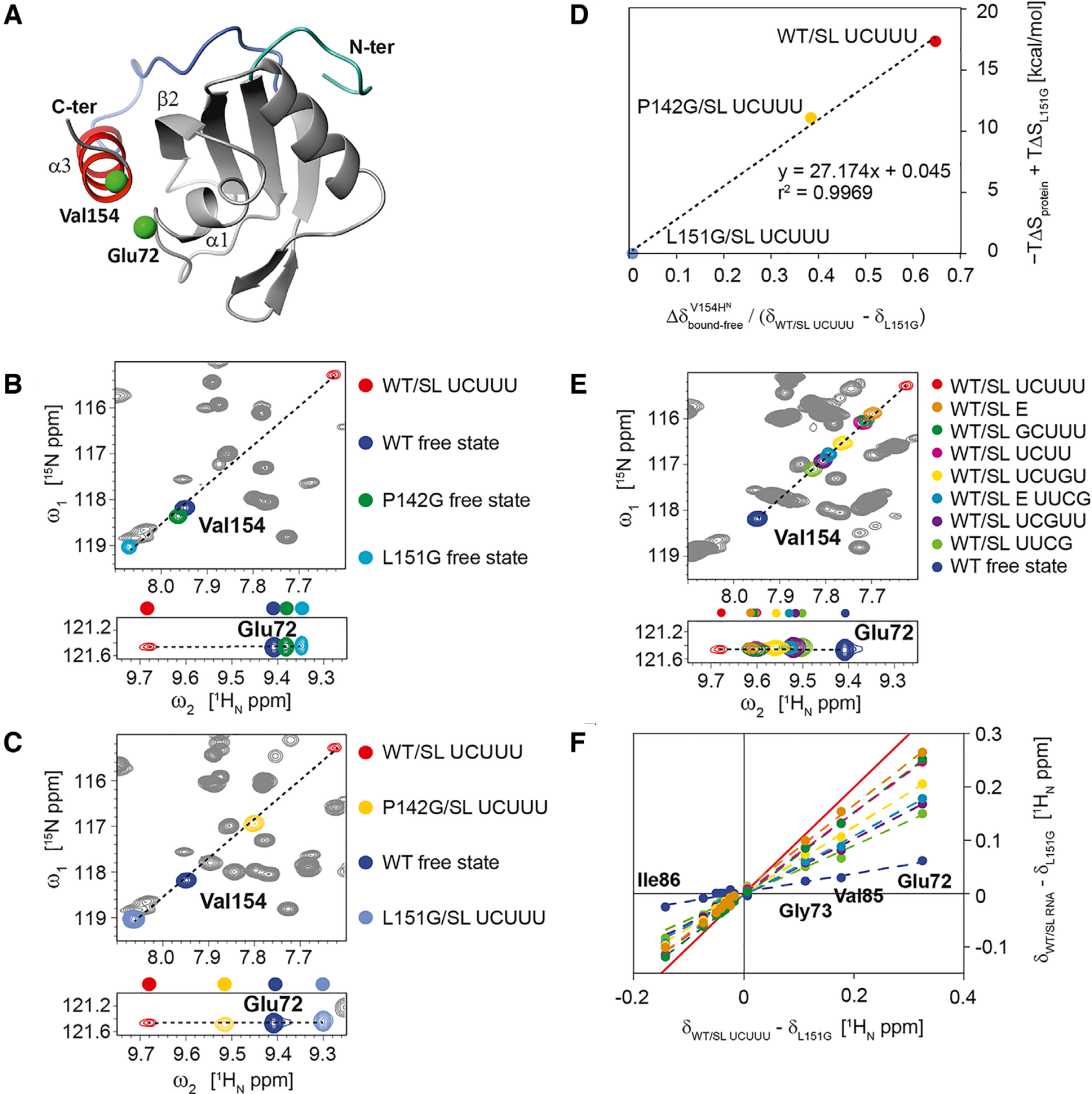
(**A**) Val154 and Glu72, residues from α3 and α1 respectively, which are monitored in ^1^H–^15^N HSQC spectra, highlighted on WT PTB RRM1/SL UCUUU complex structure. (B, C) Superposition of ^1^H-^15^N HSQC spectra of WT PTB RRM1 in the free state with (**B**) spectra of WT PTB RRM1/SL UCUUU complex and mutants P142G and L151G in the free state, (**C**) spectra of SL UCUUU-bound states of WT PTB RRM1, P142G and L151G. (**D**) Correlation of the magnitude of the amide ^1^H shift change of Val154 upon binding SL UCUUU, for WT PTB RRM1, P142G, and L151G relative to the range spanned by PTB RRM1/UCUUU and L151G, versus the entropic contribution to binding free energy relative to L151G/SL UCUUU. (**E**) ^1^H–^15^N HSQC spectra of complexes of WT PTB RRM1/SL UCUUU, WT PTB RRM1/SL E RNA and complexes of WT PTB RRM1 with various RNA loop mutants of SL UCUUU. (**F**) The ^1^H amide chemical shift difference between WT PTB RRM1/SL UCUUU complex and L151G mutant for residues 72–86 (α1–β2 segment) plotted versus shift difference between WT PTB RRM1, or its complexes with SL RNA mutants, and L151G. A similar analysis was performed with ^15^N chemical shifts and is shown in Supplementary Figure S10. The color code is the same as that employed to represent different samples in the ^1^H–^15^N HSQC spectra in (**E**). Fitting parameters for the linear least squares fitting in (E) and Supplementary Figure S10 are given in Supplementary Table S2. The red line represents a slope of 1 and corresponds to the maximum effect of α3-RRM docking observed for the WT PTB RRM1/SL UCUUU complex.

A comparison of the ^1^H–^15^N-HSQC spectra of RRM1 free and bound samples, and of the mutant L151G revealed that the crosspeaks of many amides in these samples are located on a straight line with the resonance of RRM1 free located between the L151G mutant and the RRM1 complex (Supplementary Figure S7). For example, residues Val154 and Glu72 are far from the RNA binding site and their amide signal positions are expected to be sensitive to α3 helix folding and its packing against α1 of the RRM, respectively based on their location in the structure (Figure 6A). The observed co-linear ^1^H–^15^N cross-peak positions for these resonances are consistent with the presence of a fast conformational equilibrium with respect to the chemical shift timescale among states of the C-terminus with varying degrees of helicity, which is influenced by the mutations. In the subsequent analysis, we used the magnitude of these shifts to characterize the level of α3 helix formation for different mutants of the free protein and its complexes. In addition to the amide resonance of Glu72, a number of other residues in the α1–β2 segment (which contacts α3 in the complex) show a similar pattern (Supplementary Figure S7). The position of the RRM1 free resonance is consistently between that of L151G, and RRM1 bound to SL UCUUU, suggesting that α3 helix folding in the free protein is accompanied by the formation of transient contacts with the RRM domain. The presence of a partially folded α3 helix in WT RRM1 inferred from these data suggests that complex formation occurs by a conformational selection mechanism with SL UCUUU preferentially binding to PTB RRM1 with a well folded α3. However, the presence of a C-terminal helix is not essential for stem loop RNA binding since ITC data showed that L151G can bind RNA with an affinity only two-fold weaker than the WT (Figure 4).

A comparison of the ^1^H–^15^N HSQC spectra of the L151G mutant of PTB RRM1 in the free state, and bound to SL UCUUU, clearly shows that the amide resonance of Val154 is unchanged by RNA binding. The chemical shift mapping data confirm that the α3 helix does not fold even in the presence of RNA (Figures 6B and C, Supplementary Figures S8A and S9A). In contrast, for the P142G mutant, SL UCUUU complex formation results in a shift in the position of Val154 consistent with increased helicity, in agreement with the conformational selection hypothesis (compare Figures 6B and C, or Supplementary Figures S7 and S8A). These data also suggest that the strong decrease in entropy and increase in enthalpy of binding when comparing SL UCUUU binding to L151G, P142G, and WT complexes (Figure 4) originates primarily from the increasing degree of α3 helix folding that we see in these different complexes. This relationship between the measured thermodynamics and the helical content is even quantitatively demonstrated in Figure 6D since the ^1^H chemical shift change in the three complexes which senses the helical content (e.g. Val154 amide) is proportional to the entropy change upon complex formation.

### The C-terminal helix acts as a sensor of the RNA secondary structure

We next examined the behavior of the α3 helix when RRM1 binds SL RNAs with various loop mutations. As observed for the different PTB RRM1 mutants, many signals in the complexes of PTB RRM1 with mutant SL RNA are located on a line between the signal of L151G and PTB RRM1/SL UCUUU (Supplementary Figures S8B, S9B and Figure 6E). From this we can estimate the level of α3 helix formation by correlating the size of the shift of the amide of many reporter resonances compared to L151G (no helix, 0%) and PTB RRM1/SL UCUUU (100%, Figure 6F, Supplementary Figure S10, Supplementary Tables S2 and S3). The free protein has a slope of 17% confirming the presence of a partially formed helix α3. In all the stoichiometric complexes between the WT RRM1 and the mutant RNAs, the slope is higher than 17% indicating that the degree of α3 helix content increases upon binding every SL RNA loop mutant even when the UCUUU loop is replaced by a stable UUCG tetraloop (slope of 49%, Supplementary Table S2), which as will be shown below impairs IRES function. For this latter RNA, the tetraloop structure is conserved upon binding, as indicated by the nearly identical peak positions for corresponding pyrimidine TOCSY peaks of the free and protein bound states (Supplementary Figure S9C). This suggests that the bases of the tetraloop remain tightly folded in the complex, and that binding is primarily driven by recognition of the stem–loop structure. Despite the very similar binding affinities compared to the WT UCUUU loop, none of the RNA loop mutants reaches the level of helix formation found in the complex with SL UCUUU (Figure 6E and F. Supplementary Tables S2 and S3). This indicates that among the ensemble of PTB RRM1 conformations present in solution, those with a pre-folded α3 helix are preferentially bound by all these stem-loop RNAs to varying degrees. In contrast to binding with the SL UCUUU, the binding of CUCUCU ssRNA induces more modest amide shift changes (Supplementary Figure S11A) and this is consistent with a smaller increase in ^13^C secondary shifts of the α3-region (Supplementary Figure S11B). Based on our results, we conclude that the requirements for forming a fully folded α3 helix upon RNA binding are the presence of a stem and an optimal loop sequence containing a sufficiently long polypyrimidine tract (e.g. UCUUU). To test whether a larger loop including the optimal CUU sequence bound by PTB RRM1 could promote the formation of the α3 helix to a similar extent, we compared ^15^N–^1^H HSQC spectra of the protein bound to either SL UCUUU or to hsa-mir-136 stem–loop RNA. The latter RNA contains a 9-nucleotide loop incorporating a UUCUU motif at the 5′ end of the stem (Supplementary Figure S12) (58). The positions of signals from Val154 and Glu72 closely coincide in the two HSQCs indicating the α3 helix is formed to a similar extent with the larger RNA loop. In addition, we tested RRM1 binding to SL E of the EMCV-IRES. This stem loop has also been described as an RRM1 binding site (35) and embeds the larger loop sequence UUGUCUAU. The chemical shifts of Val154 and Glu72 (Figure 6E) indicate a lower extent of α3 helix formation than for SL UCUUU, however, the analysis of the chemical shift perturbation data reveals that the level of helix formation still reaches an 84% slope (Figures 6F, Supplementary Figure S10, Supplementary Tables S2 and S3). Again, when the loop of SL E is replaced by a UUCG tetraloop, the slope showed a pronounced decrease, dropping to 57%. Altogether, these data reveal that the α3 helix of PTB RRM1 acts as a sensor allowing substrate discrimination between stem-loop containing RNA and single-stranded RNA. These data also indicate that affinity is not the only factor important for PTB to discriminate substrates since the degree of α3 helix formation clearly varies between the RNA loop sequences.

### Role of the C-terminal helix for PTB function in enhancing EMCV IRES activity

**Figure 7.**
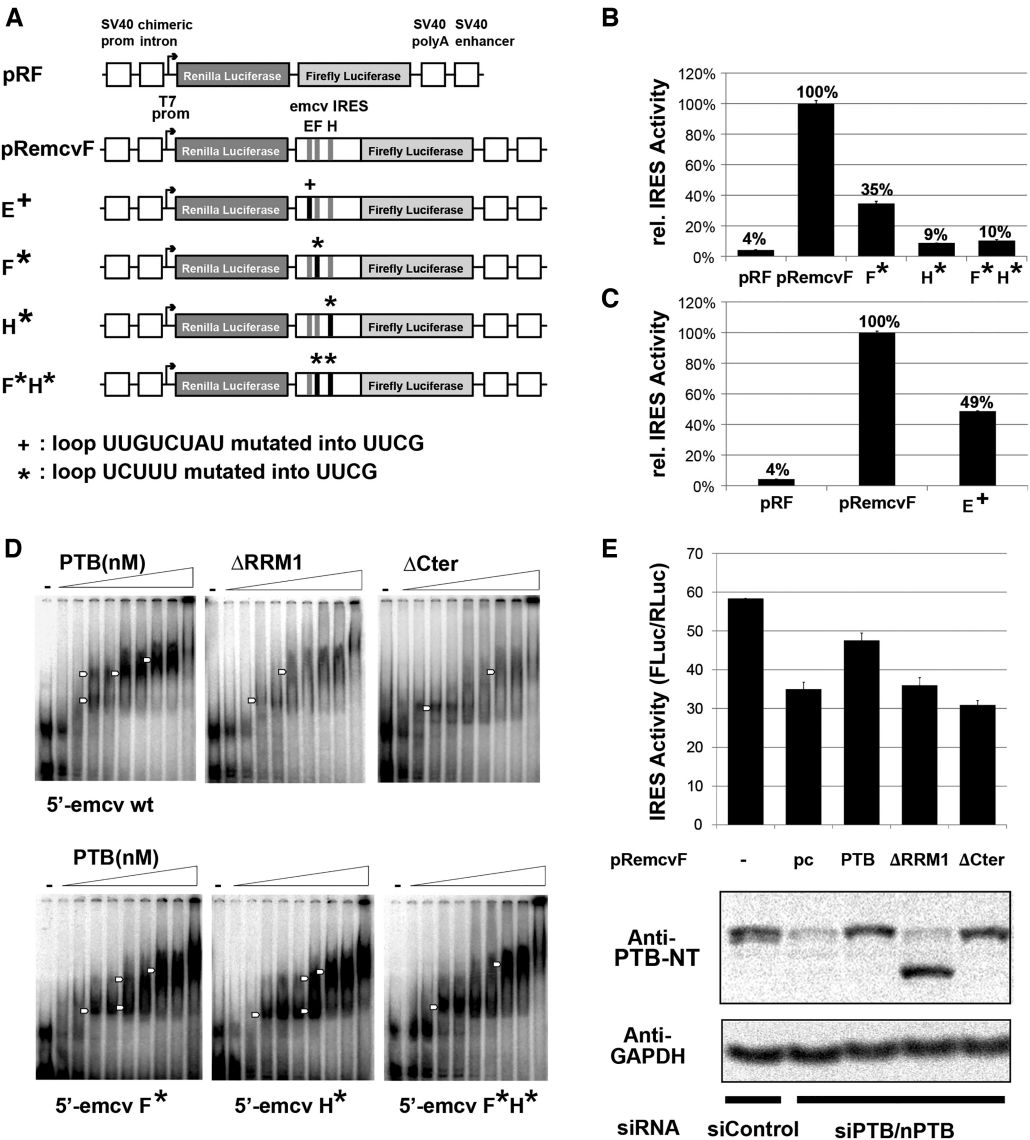
(**A**) Schematic diagram of the reporter construct pRF, the emcv IRES-containing construct pRemcvF, and the same construct with a single mutated loop E^+^, F*, H* or two mutated loops F*H*. These constructs were transfected into HEK293T cells. (**B**) IRES activity of wild type emcv and emcv IRESs with mutations of SL F, H or both F and H. Bars represent the average of two replicates ± s.d. of the IRES activity calculated as a ratio between *Firefly* and *Renilla* luciferase normalized to IRES activity of pRemcvF. (**C**) IRES activity for wt emcv compared to emcv with mutated SL E. Bars represent average values of biological duplicates, each as technical triplicate. (**D**) Upper panel: EMSA was performed using radiolabeled wild type 5′-emcv and variable concentration of wild type or mutant his-tagged PTB1. Lower panel: EMSA with radiolabeled F*, H* or F*H* mutant 5′-emcv and variable concentration of wild type his-tagged PTB1. In all EMSAs concentration of PTB1 ranged from 0, 125, 250, 375, 500, 750, 1000, 1500, 2000 and 4000 nM and complex formation is marked with white arrows. (**E**) IRES activity of wild type and mutated PTB1. Top panel: pRemcvF was transfected into HEK293T cells together with only control siRNA, or with siRNA against PTB/nPTB and either empty plasmid (pc) or plasmid expressing wild type or mutant PTB1. IRES is normalized to the IRES activity measured for pRF. Bottom panel: Equivalent amount of lysates from transfected cells was analyzed by immunoblot probed with anti-PTB-NT or anti GAPDH (loading control). This showed knockdown of endogenous PTB1 using siPTB/nPTB and similar expression levels for ectopic wild type and mutant PTB1.

To assess the role of the RRM1 C-terminal helix, in the context of the full length PTB, we compared the IRES activities of WT and mutant forms of PTB by *in vivo* translation assays using a dicistronic luciferase reporter containing an EMCV IRES as previously described (59). First, we mutated the apical loop sequence UCUUU in SL F and SL H (both separately and together) as well as the SL E UUGUCUAU sequence of EMCV IRES into the highly stable UUCG tetraloop to examine the functional importance of these loops for IRES activity (Figure 7A) (60,61). All three apical loops were previously shown to be potential PTB binding sites (27,32,35). Introducing the tetraloop mutations substantially decreased IRES activity: 2-fold for mutation in SL E, 3.6-fold for mutation in SL F and 11-fold for mutation in SL H (Figure 7B and C). Simultaneous substitution of both SL F and SL H by the tetraloop had the same effect as substitution of SL H alone. This demonstrates that the apical loop sequences in SL E, SL F and SL H are important *cis*-acting elements for EMCV IRES activity.

To evaluate the effect of the apical loop mutations on PTB binding to EMCV IRES RNA, we performed electromobility shift assays (EMSAs) (Figure 7D). We used a radiolabeled construct of EMCV IRES RNA encompassing domains D to H (169 nts) that contains binding sites for at least one PTB molecule (32). The EMSA with wild type IRES RNA and wild type PTB protein shows multiple weak bands on the gel with intermediate mobility between free and protein-bound forms which shift toward lower mobility with increasing PTB amounts reaching a plateau, indicating saturation of the IRES binding sites (Figure 7D, top left). The UUCG mutation of one or both UCUUU pentaloops (SL F and SL H) increases the intensity of the intermediate bands indicating the formation of several complexes of different size and/or conformation (Figure 7D, bottom). This suggests that a unique PTB-EMCV IRES RNA complex is favored by the presence of the UCUUU pentaloops in SL F and SL H. To probe the importance of RRM1, and in particular the region corresponding to RRM1 α3 for IRES binding, we deleted RRM1 from the full length PTB construct or only the sequence corresponding to the C-terminal α3 helix region and performed EMSAs (Figure 7D, top right panels). For both PTB mutants, the band of the complex appears as a smear due to the weaker affinity of these complexes. Thus although α3 was shown to be distal to the stem–loop binding site, its removal none-the-less impacts affinity of PTB-IRES complexes.

To assess which regions of PTB are important for EMCV IRES activity, we co-transfected FLAG-PTB expression vector containing either WT or one of the mutant forms of PTB together with the wild type luciferase dicistronic vector. To suppress the effect of the endogenous PTBs, we simultaneously knocked down endogenous PTB and nPTB using siRNA targeting their 3′-UTR (Figure 7E). As previously observed by Ventakatesan *et al.* (59), these cellular assays show that PTB/nPTB knockdown reduces EMCV IRES activity 1.6-fold and that re-introduction of PTB can partially recover the IRES activity whereas PTB without RRM1 cannot. More importantly, the deletion of the C-terminal α3 helix of RRM1 alone abrogates the IRES activity of PTB. In summary, these results show that the apical loop sequences from SL E, SL F and SL H are *cis*-acting elements of EMCV IRES. Moreover, the α3 helix-forming segment is required by PTB for optimal IRES-mediated translation, since both the deletion of the α3 segment and the replacement of the RNA loops by the UUCG tetraloop, which exhibited a low level of helix formation (Figure 6E and F), result in decreased IRES activity.

## DISCUSSION

**Figure 8.**
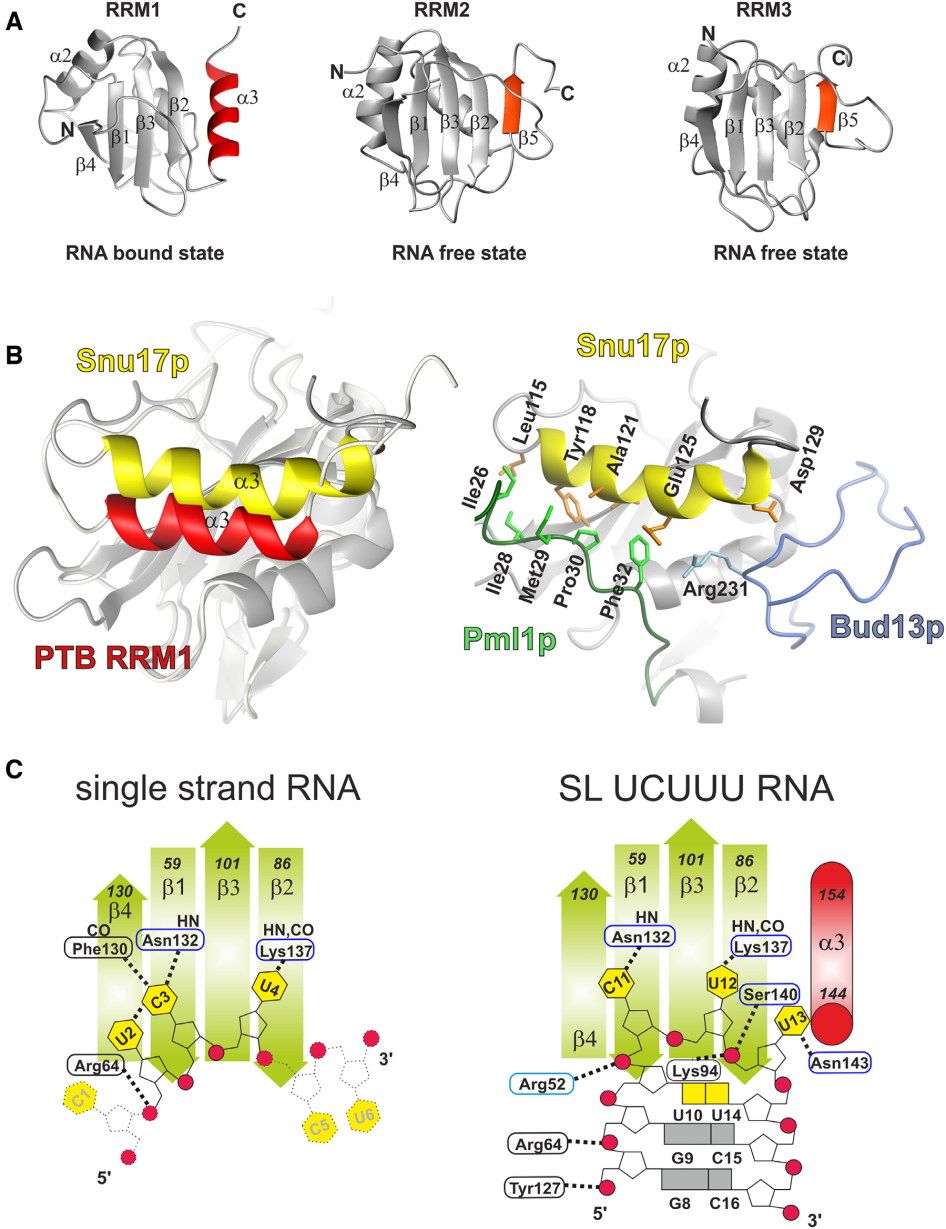
(**A**) Comparison of the structure of PTB RRM1 in the SL RNA bound state with PTB RRM2 and RRM3 in the unbound state. The additional C-terminal elements are highlighted; the α3 helix of RRM1 and the fifth β strand of RRM2 and RRM3 (accession code: 1SJR.pdb (RRM2) and 2EVZ.pdb (RRM3). (**B**) Structures of PTB RRM1 and Snup17 RRM superimposed on the RRM domain to highlight the relative position of their α3 helices, (left) and the position of the binding partners of Snu17p, (right) (accession code: 2MKC.pdb). (**C**) Schematic representation of PTB RRM1 bound to CU6-mer RNA and to SL UCUUU RNA.

The structure of PTB RRM1 in complex with SL UCUUU RNA unexpectedly revealed the presence of α3, an additional secondary structure element extending the canonical RRM motif. Previous structural work on the free RRM1 and its complex with CUCUCU RNA did not detect this helix although the RRM construct (residues 49–163) included the same C-terminal segment possibly due to the lower induced helical propensity (14,15). Based upon ^13^C secondary shifts of the RRM1 construct studied here (residues 41–163) we have shown that this α3 helix is transiently present in the free state of the protein with moderate helical character induced on binding CUCUCU single-stranded RNA (Supplementary Figure S11) whereas a strong enhancement occurs upon binding to SL UCUUU (Figure 5B). Together these data demonstrate the folding of a novel C-terminal α3 helix upon hairpin binding, and suggest that stem–loop RNA binding occurs by conformational selection, whereby the well-folded helix is most highly populated when a SL RNA with optimal loop sequence binds. The transient nature of the α3 helix implies a dynamic character, and suggest the presence of a partially ordered helix already in the free protein and we intend to study the dynamics of this system in more detail in future work. Remarkably, the α3 helix of PTB RRM1 bound to the RNA stem–loop is found in the same position as a fifth β strand found in PTB RRM2 and RRM3 which is present both in the free state as well as the RNA-bound state (Figure 8A) (15,62). This illustrates once more the critical role of the regions immediately outside the RRM for function as well as the great diversity of structural extensions that are found in RRMs (17).

Previous structural studies reported the presence of an additional helix C- or N-terminal to an RRM in the free state of RRM-containing proteins (17), as well as the displacement of a C-terminal helix upon RNA binding for the U1A N-terminal RRM (63–65). Unfolding of a C-terminal helix upon RNA binding was also observed (66). Recently it was shown that hnRNP A1, similar to PTB, contains an RRM whose binding to a cognate RNA stabilizes a C-terminal helix (67). However, unlike PTB, the RRM of hnRNP A1 is part of a tandem pair that forms a joint RNA-binding interface and the C-terminal helix is oriented perpendicular to the β-sheet strands, and makes contacts with the RNA, which is single-stranded. PTB RRM1 is the first example to our knowledge in which folding of a C-terminal helix occurs in an RRM upon binding to a structured RNA. Our RRM1–RNA complex presents interesting parallels with the recent solution structure of a ternary protein complex between the RRM of Snup17p, and two peptides from Bud13p and Plm1p (68). The C-terminal extension of Snup17p RRM, which is unstructured in the free state folds into a four-turn helix α3 upon binding of the two peptides. Snup17p helix α3 adopts a position very similar but not identical to the one found in PTB RRM1–SL UCUUU complex (Figure 8B). In contrast to the helix α3 of PTB RRM1, which has no direct contact to the RNA, the α3 helix of Snup17 interacts extensively with Plm1p and also contacts Bud13p. Together these findings show that both the binding of proteins and structured RNAs can act as triggers for the formation of additional RRM structural elements (15). PTB RRM1 binds a single stranded RNA much more weakly than stem–loop RNA, with a dissociation constant of 25 μM, which is 250-fold higher than that of the hairpin complex (30). Quite surprisingly, our study revealed that the presence of a stem–loop is more important than the RNA sequence itself for the increased affinity, since changing the sequence of the loop to the UUCG tetraloop decreases the affinity only 4-fold. Comparing the structures of the hairpin and single-strand complexes reveals both similarities in the interactions and differences, which help to explain the enhanced affinity for stem–loop RNA. In both cases, the CU dinucleotide is arranged similarly on the β-sheet surface but the recognition of the Watson–Crick face of the C is less pronounced in the stem–loop (Figure 8C). Upstream of the C11–U12 motif in the stem–loop complex, the U10 nucleotide forms a mismatched base pair with U14. This UU basepair forms important stacking contacts with Leu94 in the β2-β3 loop, which also makes ionic interactions with the phosphate backbone of U13. In contrast, U2 the nucleotide upstream of the CU motif in the single strand complex, is positioned over β4 and stacks with the Arg64 sidechain which also makes nonspecific interactions with the U2 phosphate. In the hairpin complex, the side chain of Arg52 from the region N-terminal to the RRM interacts with the C11 phosphate, whereas it does not interact with C3 in the complex with single-stranded RNA (Figure 8C). Additionally, Tyr127 contacts the phosphate of G8 in the stem–loop whereas there is no corresponding nucleotide in the single-stranded complex. U13, one nucleotide downstream of the CU motif in the stem–loop, is positioned at the end of the β2 strand and stabilizes the region just upstream of the α3 helix by interacting with Ser140 and Asn143, whereas the corresponding nucleotide C5 in the complex with the single stranded RNA is unstructured.

The many contacts from PTB RRM1 to the phosphate backbone help to recognize the shape of the RNA and partially account for the increased affinity for stem–loops (Figure 8C). These contacts are concentrated at the 5′ end of the stem (G8 and G9), and the similar binding behavior of hairpins containing larger loops such as SL E and hsa-mir-136 stem–loop RNA suggests that RRM1 anchors to the stem via these intermolecular contacts. Moreover, superposition of the UUCG tetraloop structure (69) onto the coordinates of SL UCUUU assuming a similar arrangement, shows that many of these nonspecific intermolecular contacts can be accommodated (Supplementary Figure S13) and this may explain the relatively moderate impact of substituting the UUCG tetraloop on PTB RRM1 binding (Figure 4). Overall, the role of RRM1 in PTB might therefore be to specifically target stem–loop RNA. This would rationalize earlier findings suggesting that the N-terminal RRMs (RRM1 and RRM2) prefers U-tracts embedded in stem-loops while the C-terminal RRMs prefer single-stranded RNA (34).

Although RRM1 shows only modest differences in affinity for different pyrimidine-rich loop sequences when binding a structured RNA, these sequence differences do impact α3 helix formation. This may be related to the degree to which the apical loop bases can form interactions, which help to stabilize the position of the RRM1 β4–α3 loop on the β-sheet and organize the binding pocket for U13 just N-terminal to α3. In this context the high stability of the UUCG loop may prevent conformational adaption of the RRM1 which help to stabilize α3. Our *in vivo* data demonstrated that the helix α3 is essential for PTB in promoting EMCV IRES activity, and that the E, F and H apical loops, which are PTB binding sites, are crucial cis-acting elements for EMCV IRES (Figure 7). It has been shown that PTB facilitates the functional conformation of viral IRES sequences and acts as an RNA chaperone (4,33,70). It was surprising to find that mutating one or two of the apical loops into UUCG could impair IRES activity since RRM1 shows only a 4-fold decrease in binding affinity for SL UUCG over SL UCUUU. However, one major difference between the two complexes resides in the level of folding of the α3 helix which is significantly lower in the SL UUCG complexes than in the SL UCUUU complex or the SL E complex (Figure 6E, F and Supplementary Figure S9B). We can therefore hypothesize that even though RRM1 can bind to the RNA, PTB is able to remodel EMCV IRES RNA only if the α3 helix is fully folded. This may partly explain the increased heterogeneity in EMSAs of the PTB/IRES complexes where either α3 is deleted or a stem–loop is substituted (Figure 7D). PTB RRM1 is the first example of an RRM transducing the recognition of an RNA, on the basis of secondary structure and sequence, into the folding of an additional structural element (α3) which is distant from the binding site, thus representing an allosteric mechanism for controlling PTB function. The role of helix α3 may be to favor a defined orientation between RRM1 and RRM2, since the folding of the α3 helix rigidifies part of the linker between RRM1 and RRM2. This could facilitate the formation of a productive conformation for binding of eukaryotic initiation factors, or other IRES trans acting factors. It remains to be seen if the folding of helix α3 of RRM1 is important in alternative splicing where the RNA secondary structure also plays an important role (71), either for the binding of PTB to cis-acting elements or spliceosome components (30).

## DATA AVAILABILITY

Chemical shifts and atomic coordinates of the PTB RRM1/SL UCUUU complex have been deposited under accession codes BMRB 25652 and PDB 2N3O respectively.

## Supplementary Material

gkaa155_Supplemental_File

## Notes

Present address: Sandrine Jayne, Leicester Cancer Research Centre, University of Leicester, Leicester, UK.

Present address: Irene Beusch, Department of Biochemistry and Biophysics, University of California, San Francisco, CA 94158, USA.

## References

[1] Dominguez D., Freese P., Alexis M.S., Su A., Hochman M., Palden T., Bazile C., Lambert N.J., Van Nostrand E.L., Pratt G.A. et al. Sequence, structure, and context preferences of human RNA binding proteins. Mol. Cell. 2018; 70:854–867. 29883606 10.1016/j.molcel.2018.05.001 PMC6062212

[2] Sawicka K., Bushell M., Spriggs K.A., Willis A.E. Polypyrimidine-tract-binding protein: a multifunctional RNA-binding protein. Biochem. Soc. Trans. 2008; 36:641–647. 18631133 10.1042/BST0360641

[3] Kafasla P., Mickleburgh I., Llorian M., Coelho M., Gooding C., Cherny D., Joshi A., Kotik-Kogan O., Curry S., Eperon I.C. et al. Defining the roles and interactions of PTB. Biochem. Soc. Trans. 2012; 40:815–820. 22817740 10.1042/BST20120044

[4] Romanelli M.G., Diani E., Lievens P.M. New insights into functional roles of the polypyrimidine tract-binding protein. Int. J. Mol. Sci. 2013; 14:22906–22932. 24264039 10.3390/ijms141122906 PMC3856098

[5] Garcia-Blanco M.A., Jamison S.F., Sharp P.A. Identification and purification of a 62,000-Dalton protein that binds specifically to the polypyrimidine tract of introns. Genes Dev. 1989; 3:1874–1886. 2533575 10.1101/gad.3.12a.1874

[6] Llorian M., Schwartz S., Clark T.A., Hollander D., Tan L.Y., Spellman R., Gordon A., Schweitzer A.C., la Grange P., Ast G. et al. Position-dependent alternative splicing activity revealed by global profiling of alternative splicing events regulated by PTB. Nat. Struct. Mol. Biol. 2010; 17:1114–1123. 20711188 10.1038/nsmb.1881 PMC2933513

[7] Lou H., Helfman D.M., Gagel R.F., Berget S.M. Polypyrimidine tract-binding protein positively regulates inclusion of an alternative 3 '-terminal exon. Mol. Cell Biochem. 1999; 19:78–85. 10.1128/mcb.19.1.78 PMC83867 9858533

[8] Castelo-Branco P., Furger A., Wollerton M., Smith C., Moreira A., Proudfoot N. Polypyrimidine tract binding protein modulates efficiency of polyadenylation. Mol. Cell Biochem. 2004; 24:4174–4183. 10.1128/MCB.24.10.4174-4183.2004 PMC400487 15121839

[9] Wollerton M.C., Gooding C., Wagner E.J., Garcia-Blanco M.A., Smith C.W.J. Autoregulation of polypyrimidine tract binding protein by alternative splicing leading to nonsense-mediated decay. Mol. Cell. 2004; 13:91–100. 14731397 10.1016/s1097-2765(03)00502-1

[10] Song Y.T., Tzima E., Ochs K., Bassili G., Trusheim H., Linder M., Preissner K.T., Niepmann M. Evidence for an RNA chaperone function of polypyrimidine tract-binding protein in picornavirus translation. RNA. 2005; 11:1809–1824. 16314455 10.1261/rna.7430405 PMC1370870

[11] Chang K.S., Luo G.X. The polypyrimidine tract-binding protein (PTB) is required for efficient replication of hepatitis C virus (HCV) RNA. Virus Res. 2006; 115:1–8. 16102869 10.1016/j.virusres.2005.06.012

[12] Jang S.K., Wimmer E. Cap-Independent translation of encephalomyocarditis virus-RNA - structural elements of the internal ribosomal entry site and involvement of a cellular 57-Kd RNA-Binding protein. Genes Dev. 1990; 4:1560–1572. 2174810 10.1101/gad.4.9.1560

[13] Petoukhov M.V., Monie T.P., Allain F.H.T., Matthews S., Curry S., Svergun D.I. Conformation of polypyrimidine tract binding protein in solution. Structure. 2006; 14:1021–1027. 16765895 10.1016/j.str.2006.04.005

[14] Simpson P.J., Monie T.P., Szendroi A., Davydova N., Tyzack J.K., Conte M.R., Read C.M., Cary P.D., Svergun D.I., Konarev P.V. et al. Structure and RNA interactions of the N-terminal RRM domains of PTB. Structure. 2004; 12:1631–1643. 15341728 10.1016/j.str.2004.07.008

[15] Oberstrass F.C., Auweter S.D., Erat M., Hargous Y., Henning A., Wenter P., Reymond L., Amir-Ahmady B., Pitsch S., Black D.L. et al. Structure of PTB bound to RNA: Specific binding and implications for splicing regulation. Science. 2005; 309:2054–2057. 16179478 10.1126/science.1114066

[16] Li B., Yen T.S.B. Characterization of the nuclear export signal of polypyrimidine tract-binding protein. J. Biol. Chem. 2002; 277:10306–10314. 11781313 10.1074/jbc.M109686200

[17] Maris C., Dominguez C., Allain F.H.T. The RNA recognition motif, a plastic RNA-binding platform to regulate post-transcriptional gene expression. FEBS J. 2005; 272:2118–2131. 15853797 10.1111/j.1742-4658.2005.04653.x

[18] Vitali F., Henning A., Oberstrass F.C., Hargous Y., Auweter S.D., Erat M., Allain F.H.T. Structure of the two most C-terminal RNA recognition motifs of PTB using segmental isotope labeling. EMBO J. 2006; 25:150–162. 16362043 10.1038/sj.emboj.7600911 PMC1356354

[19] Lamichhane R., Daubner G.M., Thomas-Crusells J., Auweter S.D., Manatschal C., Austin K.S., Valniuk O., Allain F.H.T., Rueda D. RNA looping by PTB: Evidence using FRET and NMR spectroscopy for a role in splicing repression. Proc. Natl. Acad. Sci. U.S.A. 2010; 107:4105–4110. 20160105 10.1073/pnas.0907072107 PMC2840148

[20] Wollerton M.C., Gooding C., Robinson F., Brown E.C., Jackson R.J., Smith C.W.J. Differential alternative splicing activity of isoforms of polypyrimidine tract binding protein (PTB). RNA. 2001; 7:819–832. 11421360 10.1017/s1355838201010214 PMC1370133

[21] Ghetti A., Pinolroma S., Michael W.M., Morandi C., Dreyfuss G. HnRNP-I, the polypyrimidine tract-binding protein - distinct nuclear localization and association with HnRNAs. Nucleic Acids Res. 1992; 20:3671–3678. 1641332 10.1093/nar/20.14.3671 PMC334017

[22] Gil A., Sharp P.A., Jamison S.F., Garcia-Blanco M.A. Characterization of cDNAs encoding the polypyrimidine tract-binding protein. Genes Dev. 1991; 5:1224–1236. 1906035 10.1101/gad.5.7.1224

[23] Hamilton B.J., Genin A., Cron R.Q., Rigby W.F.C. Delineation of a novel pathway that regulates CD154 (CD40 ligand) expression. Mol. Cell Biochem. 2003; 23:510–525. 10.1128/MCB.23.2.510-525.2003 PMC151525 12509450

[24] Robinson F., Jackson R.J., Smith C.W.J. Expression of human nPTB Is limited by extreme suboptimal codon content. PLoS One. 2008; 3:e1801. 18335065 10.1371/journal.pone.0001801 PMC2258417

[25] Auweter S.D., Oberstrass F.C., Allain F.H.T. Solving the structure of PTB in complex with pyrimidine tracts: An NMR study of protein-RNA complexes of weak affinities. J. Mol. Biol. 2007; 367:174–186. 17239394 10.1016/j.jmb.2006.12.053

[26] Brown E.A., Zhang H.C., Ping L.H., Lemon S.M. Secondary structure of the 5′ nontranslated regions of hepatitis-C virus and pestivirus genomic RNAs. Nucleic Acids Res. 1992; 20:5041–5045. 1329037 10.1093/nar/20.19.5041 PMC334281

[27] Kolupaeva V.G., Hellen C.U.T., Shatsky I.N. Structural analysis of the interaction of the pyrimidine tract-binding protein with the internal ribosomal entry site of encephalomyocarditis virus and foot-and-mouth disease virus RNAs. RNA. 1996; 2:1199–1212. 8972770 PMC1369448

[28] Garlapati S., Wang C.C. Identification of a novel internal ribosome entry site in giardiavirus that extends to both sides of the initiation codon. J. Biol. Chem. 2004; 279:3389–3397. 14615487 10.1074/jbc.M307565200

[29] Garlapati S., Wang C.C. Identification of an essential pseudoknot in the putative downstream internal ribosome entry site in giardiavirus transcript. RNA. 2002; 8:601–611. 12022227 10.1017/s135583820202071x PMC1370281

[30] Sharma S., Maris C., Allain F.H.T., Black D.L. U1 snRNA directly interacts with polypyrimidine tract-binding protein during splicing repression. Mol. Cell. 2011; 41:579–588. 21362553 10.1016/j.molcel.2011.02.012 PMC3931528

[31] Kaminski A., Hunt S.L., Patton J.G., Jackson R.J. Direct evidence that polypyrimidine tract binding protein (PTB) is essential for internal initiation of translation of encephalomyocarditis virus RNA. RNA. 1995; 1:924–938. 8548657 PMC1369341

[32] Kafasla P., Morgner N., Poyry T.A.A., Curry S., Robinson C.V., Jackson R.J. Polypyrimidine tract binding protein stabilizes the encephalomyocarditis virus IRES structure via binding multiple sites in a unique orientation. Mol. Cell. 2009; 34:556–568. 19524536 10.1016/j.molcel.2009.04.015

[33] Kafasla P., Morgner N., Robinson C.V., Jackson R.J. Polypyrimidine tract-binding protein stimulates the poliovirus IRES by modulating eIF4G binding. EMBO J. 2010; 29:3710–3722. 20859255 10.1038/emboj.2010.231 PMC2982756

[34] Clerte C., Hall K.B. The domains of polypyrimidine tract binding protein have distinct RNA structural preferences. Biochemistry. 2009; 48:2063–2074. 19226116 10.1021/bi8016872 PMC2766422

[35] Dorn G., Leitner A., Boudet J., Campagne S., von Schroetter C., Moursy A., Aebersold R., Allain F.H.T. Structural modeling of protein-RNA complexes using crosslinking of segmentally isotope-labeled RNA and MS/MS. Nat. Methods. 2017; 14:487–490. 28346450 10.1038/nmeth.4235 PMC5505470

[36] Guillerez J., Lopez P.J., Proux F., Launay H., Dreyfus M. A mutation in T7 RNA polymerase that facilitates promoter clearance. Proc. Natl. Acad. Sci. U.S.A. 2005; 102:5958–5963. 15831591 10.1073/pnas.0407141102 PMC1087904

[37] Wenter P., Reymond L., Auweter S.D., Allain F.H.T., Pitsch S. Short, synthetic and selectively ^13^C-labeled RNA sequences for the NMR structure determination of protein-RNA complexes. Nucleic Acids Res. 2006; 34:e79. 16807315 10.1093/nar/gkl427 PMC1904103

[38] Keller R.L.J. The Computer Aided Resonance Assignment Tutorial. 2004; Goldau CANTINA Verlag.

[39] Cavanagh J. Protein NMR Spectroscopy: Principles and Practice. 2007; 2nd edn Amsterdam; Boston Academic Press.

[40] Wohnert J., Ramachandran R., Gorlach M., Brown L.R. Triple-resonance experiments for correlation of H5 and exchangeable pyrimidine base hydrogens in ^13^C, ^15^N-labeled RNA. J. Magn. Reson. 1999; 139:430–433. 10423381 10.1006/jmre.1999.1797

[41] Kjaergaard M., Poulsen F.M. Sequence correction of random coil chemical shifts: correlation between neighbor correction factors and changes in the Ramachandran distribution. J. Biomol. NMR. 2011; 50:157–165. 21604143 10.1007/s10858-011-9508-2

[42] Kjaergaard M., Brander S., Poulsen F.M. Random coil chemical shift for intrinsically disordered proteins: effects of temperature and pH. J. Biomol. NMR. 2011; 49:139–149. 21234644 10.1007/s10858-011-9472-x

[43] Herrmann T., Güntert P., Wüthrich K. Protein NMR structure determination with automated NOE assignment using the new software CANDID and the torsion angle dynamics algorithm DYANA. J. Mol. Biol. 2002; 319:209–227. 12051947 10.1016/s0022-2836(02)00241-3

[44] Herrmann T., Güntert P., Wüthrich K. Protein NMR structure determination with automated NOE-identification in the NOESY spectra using the new software ATNOS. J. Biomol. NMR. 2002; 24:171–189. 12522306 10.1023/a:1021614115432

[45] Güntert P., Buchner L. Combined automated NOE assignment and structure calculation with CYANA. J. Biomol. NMR. 2015; 62:453–471. 25801209 10.1007/s10858-015-9924-9

[46] Cornilescu G., Delaglio F., Bax A. Protein backbone angle restraints from searching a database for chemical shift and sequence homology. J. Biomol. NMR. 1999; 13:289–302. 10212987 10.1023/a:1008392405740

[47] Wuttke D.S., Foster M.P., Case D.A., Gottesfeld J.M., Wright P.E. Solution structure of the first three zinc fingers of TFIIIA bound to the cognate DNA sequence: Determinants of affinity and sequence specificity. J. Mol. Biol. 1997; 273:183–206. 9367756 10.1006/jmbi.1997.1291

[48] Case D.A., T.A. D., Cheatham T.E., Simmerling C.I., Wang J., Duke R.E., Luo R., Walker R.C., Zhang W., Merz K.W. et al. AMBER12. 2012; San Francisco University of California.

[49] Oberstrass F.C., Lee A., Stefl R., Janis M., Chanfreau G., Allain F.H.T. Shape-specific recognition in the structure of the Vts1p SAM domain with RNA. Nature Struct. Mol. Biol. 2006; 13:160–167. 16429156 10.1038/nsmb1038

[50] Laskowski R.A., Rullmann J.A.C., MacArthur M.W., Kaptein R., Thornton J.M. AQUA and PROCHECK-NMR: programs for checking the quality of protein structures solved by NMR. J. Biomol. NMR. 1996; 8:477–486. 9008363 10.1007/BF00228148

[51] Cornilescu G., Marquardt J.L., Ottiger M., Bax A. Validation of protein structure from anisotropic carbonyl chemical shifts in a dilute liquid crystalline phase. J. Am. Chem. Soc. 1998; 120:6836–6837.

[52] Koradi R., Billeter M., Wüthrich K. MOLMOL: a program for display and analysis of macromolecular structures. J. Mol. Graphics. 1996; 14:51–55. 10.1016/0263-7855(96)00009-4 8744573

[53] Stoneley M., Subkhankulova T., Le Quesne J.P.C., Coldwell M.J., Jopling C.L., Belsham G.J., Willis A.E. Analysis of the c-myc IRES; a potential role for cell-type specific trans-acting factors and the nuclear compartment. Nucleic Acids Res. 2000; 28:687–694. 10637319 10.1093/nar/28.3.687 PMC102558

[54] Spellman R., Llorian M., Smith C.W.J. Crossregulation and functional redundancy between the splicing regulator PTB and its paralogs nPTB and ROD1. Mol. Cell. 2007; 27:420–434. 17679092 10.1016/j.molcel.2007.06.016 PMC1940037

[55] Daubner G.M., Clery A., Allain F.H.T. RRM-RNA recognition: NMR or crystallography … and new findings. Curr. Opin. Struct. Biol. 2013; 23:100–108. 23253355 10.1016/j.sbi.2012.11.006

[56] Spera S., Bax A. Empirical correlation between protein backbone conformation and Cα and Cβ ^13^C nuclear magnetic resonance chemical shifts. J. Am. Chem. Soc. 1991; 113:5490–5492.

[57] Wang L.Y., Markley J.L. Empirical correlation between protein backbone ^15^N and ^13^C secondary chemical shifts and its application to nitrogen chemical shift re-referencing. J. Biomol. NMR. 2009; 44:95–99. 19436955 10.1007/s10858-009-9324-0 PMC2782637

[58] Michlewski G., Guil S., Semple C.A., Caceres J.F. Posttranscriptional regulation of miRNAs harboring conserved terminal loops. Mol. Cell. 2008; 32:383–393. 18995836 10.1016/j.molcel.2008.10.013 PMC2631628

[59] Venkatesan A., Sharma R., Dasgupta A. Cell cycle regulation of hepatitis C and encephalomyocarditis virus internal ribosome entry site-mediated translation in human embryonic kidney 293 cells. Virus Res. 2003; 94:85–95. 12902037 10.1016/s0168-1702(03)00136-9

[60] Denisov A.Y., Hannoush R.N., Gehring K., Damha M.J. A novel RNA motif based on the structure of unusually stable 2′,5′-linked r(UUCG) loops. J. Am. Chem. Soc. 2003; 125:11525–11531. 13129354 10.1021/ja036207k

[61] Cheong C.J., Varani G., Tinoco I. Solution structure of an unusually stable RNA hairpin, 5′GGAC(UUCG)GUCC. Nature. 1990; 346:680–682. 1696688 10.1038/346680a0

[62] Conte M.R., Grune T., Ghuman J., Kelly G., Ladas A., Matthews S., Curry S. Structure of tandem RNA recognition motifs from polypyrimidine tract binding protein reveals novel features of the RRM fold. EMBO J. 2000; 19:3132–3141. 10856256 10.1093/emboj/19.12.3132 PMC203357

[63] Avis J.M., Allain F.H.T., Howe P.W.A., Varani G., Nagai K., Neuhaus D. Solution structure of the N-terminal RNP domain of U1A protein: The role of C-terminal residues in structure stability and RNA binding. J. Mol. Biol. 1996; 257:398–411. 8609632 10.1006/jmbi.1996.0171

[64] Oubridge C., Ito N., Evans P.R., Teo C.H., Nagai K. Crystal-structure at 1.92 angstrom resolution of the RNA-Binding domain of the U1a spliceosomal protein complexed with an RNA hairpin. Nature. 1994; 372:432–438. 7984237 10.1038/372432a0

[65] Allain F.H.T., Gubser C.C., Howe P.W.A., Nagai K., Neuhaus D., Varani G. Specificity of ribonucleoprotein interaction determined by RNA folding during complex formation. Nature. 1996; 380:646–650. 8602269 10.1038/380646a0

[66] Canadillas J.M.P., Varani G. Recognition of GU-rich polyadenylation regulatory elements by human CstF-64 protein. EMBO J. 2003; 22:2821–2830. 12773396 10.1093/emboj/cdg259 PMC156756

[67] Beusch I., Barraud P., Moursy A., Clery A., Allain F.H.T. Tandem hnRNP A1 RNA recognition motifs act in concert to repress the splicing of survival motor neuron exon 7. Elife. 2017; 6:e25736. 28650318 10.7554/eLife.25736 PMC5503513

[68] Wysoczanski P., Schneider C., Xiang S., Munari F., Trowitzsch S., Wahl M.C., Luhrmann R., Becker S., Zweckstetter M. Cooperative structure of the heterotrimeric pre-mRNA retention and splicing complex. Nat. Struct. Mol. Biol. 2014; 21:911–918. 25218446 10.1038/nsmb.2889

[69] Allain F.H.T., Varani G. Structure of the P1 helix from group-I self-splicing introns. J. Mol. Biol. 1995; 250:333–353. 7608979 10.1006/jmbi.1995.0381

[70] Yu Y.P., Abaeva I.S., Marintchev A., Pestova T.V., Hellen C.U.T. Common conformational changes induced in type 2 picornavirus IRESs by cognate trans-acting factors. Nucleic Acids Res. 2011; 39:4851–4865. 21306989 10.1093/nar/gkr045 PMC3113573

[71] McManus C.J., Graveley B.R. RNA structure and the mechanisms of alternative splicing. Curr. Opin. Genet. Dev. 2011; 21:373–379. 21530232 10.1016/j.gde.2011.04.001 PMC3149766

