# Supplementary material for: A transient α-helix in the N-terminal RNA recognition motif of polypyrimidine tract binding protein senses RNA secondary structure
Source: Nucleic Acids Res. 2020 Mar 14;48(8):4521–37. doi: 10.1093/nar/gkaa155 (PMC7192611; doi:10.1093/nar/gkaa155)

Supplementary data

A transient α-helix in the N-terminal RNA Recognition Motif of Polypyrimidine Tract Binding Protein senses RNA Secondary Structure

Christophe Maris[1,*], Sandrine Jayne[1], Fred F. Damberger[1], Irene Beusch[1], Georg Dorn[1], Sapna Ravindranathan[2] and Frédéric H.-T. Allain[1,*]

[1]Department of Biology, ETH Zurich, 8093 Zürich, Switzerland

[2]Central NMR facility, National Chemical Laboratory, Pune 411008 India

[*] Corresponding authors. allain@mol.biol.ethz.ch, christophe.j.maris@gmail.com.

Present address: Sandrine Jayne, Leicester Cancer Research Centre, University of Leicester, Leicester, United Kingdom, Irene Beusch, Department of Biochemistry and Biophysics, University of California, San Francisco, CA 94158, USA

Table S1. Structure statistics for PTB RRM1/SL UCUUU

**NMR restraints**		
Distance Restraints		2318
Protein intramolecular		1749
	intraresidual	367
	sequential ($\lvert i-j\rvert=1$)	415
	medium range ($1<\lvert i-j\rvert<5$)	377
	long range ($\lvert i-j\rvert>=5$)	562
	hydrogen bonds[a]	21
	residual dipolar couplings	40
RNA	intramolecular	471
	intraresidual	247
	sequential ($\lvert i-j\rvert=1$)	159
	medium range ($1<\lvert i-j\rvert<5$)	4
	long range ($\lvert i-j\rvert>=5$)	61
	hydrogen bonds[a]	27
	residual dipolar couplings	31
Complex	intermolecular	98
	long range ($\lvert i-j\rvert>=5$)	98
	hydrogen bonds[a]	2
Torsion angles[b]		107
RNA	sugar pucker (δ)	23
	other	84
**Structure statistics[d]**		
Average distance constraint violations		
0.1-0.2 Å		33.5 ± 4.4
0.2-0.3 Å		7.3 ± 2.1
0.3-0.4 Å		0.3 ± 0.6
>0.4 Å		0.0 ± 0.0
Maximal		0.29 ± 0.02 Å
Average angle constraint violations		
<5°		28.1 ± 1.8
>5°		0.5 ± 0.6
Maximal		4.72 ± 1.68 °
Mean Deviation from ideal covalent geometry[e]		
Bond Length		0.0039 ± 0.0000 Å
Bond Angle		1.732 ± 0.021 °
Residual dipolar coupling constraints		
Q factor[c]		0.28
Absolute violation		0.2 ± 1.6 Hz
**Ramachandran plot statistics[d,e,f]**		
Residues in most favored regions		87.3 ± 2.3 %
Residues in additionally allowed regions		11.8 ± 2.5 %
Residues in generously allowed regions		0.5 ± 0.9 %
Residues in disallowed regions		0.4 ± 0.5 %
**RMSD to mean structure statistics[d]**		
Protein (58-155)		
Backbone atoms		0.45 ± 0.06 Å
Heavy atoms		0.83 ± 0.10 Å
RNA (1-23)		
Backbone atoms		0.66 ± 0.25 Å
Heavy atoms		0.66 ± 0.23 Å
All molecules		
Backbone atoms		1.08 ± 0.48 Å
Heavy atoms		1.22 ± 0.44 Å

[a] For the protein, hydrogen bond restraints were implemented for those amide protons protected from solvent exchange. For the RNA, hydrogen bond restraints were implemented for the experimentally identified RNA base pairs.

[b] For the protein, dihedral angle restraints were generated by TALOS on the basis of backbone atom chemical shifts and by analysis of local NOE patterns. For the RNA, the sugar pucker was determined based on homonuclear TOCSY. Other dihedral angle restraints (α, β, ε, γ and ζ angle) were generated based on the NOE pattern typical of A form RNA helix.

[c] Q factor is normalized to square deviation.

[d] Statistics computed for the deposited bundle of 20 structures with lowest violation energy selected from the 30 structures with lowest amber energy.

[e] as defined by the program PROCHECK-NMR.

Table S2. Degree of docked α3 helix in PTB RRM1 free & bound complexes relative to WT/SL UCUUU

Least squares fit of data presented in Fig. 6E and in Fig. S10A (using ^{15}N shifts) to estimate the relative amount of docked helix for various states of PTB RRM1 (free and bound to mutant SL RNAs) compared to the PTB RRM1/SL UCUUU complex. Column 1 is the slope of data from ^{1}H (Fig. 6E), column 2 is the Pearson coefficient of the fit, column 3 is the slope of the data for ^{15}N (Fig. S10A), column 4 is the Pearson coefficient for ^{15}N data, and column 5 is the average and standard deviation of the slopes for ^{1}H and ^{15}N data of the same PTB RRM1 state.

Condition	$f(^{1}H)$	$r^2(^{1}H)$	$f(^{15}N)$	$r^2(^{15}N)$	f^{ave}
free PTB RRM1	0.16	0.92	0.18	0.94	0.17±0.01
PTB RRM1/SL UCUUU variants					
PTB RRM1/SL **G**CUUU	0.78	0.9996	0.79	0.9992	0.79 ± 0.01
PTB RRM1/SL UCUU_	0.76	0.999	0.79	0.9993	0.78 ± 0.02
PTB RRM1/SL UCU**G**U	0.64	0.997	0.67	0.998	0.66 ± 0.02
PTB RRM1/SL UC**G**UU	0.53	0.988	0.56	0.996	0.55 ± 0.02
PTB RRM1/SL UUCG	0.46	0.98	0.51	0.991	0.49 ± 0.04
PTB RRM1/SLE variants					
PTB RRM1/SL E	0.81	0.996	0.86	0.998	0.84 ± 0.04
PTB RRM1/SL E UUCG	0.56	0.993	0.58	0.996	0.57 ± 0.01

Table S3. Degree of folded PTB RRM1 α3 helix in free and bound SL complexes relative to WT/SL UCUUU

Least squares fit of data presented in Fig. S10B and C to estimate the relative amount of helix formed for various states of PTB RRM1 (free and bound to mutant SL RNAs) compared to the PTB RRM1/SL UCUUU complex. Column 1 is the slope of data from ^{1}H (Fig. S10B), column 2 is the Pearson coefficient of the fit, column 3 is the slope of the data for ^{15}N (Fig. S10C), column 4 is the Pearson coefficient for ^{15}N data, and column 5 is the average and standard deviation of the slopes for ^{1}H and ^{15}N data of the same PTB RRM1 state.

Condition	$f(^{1}H)$	$r^2(^{1}H)$	$f(^{15}N)$	$r^2(^{15}N)$	f^{ave}
free PTB RRM1	0.2232	0.8306	0.185	0.6155	0.20±0.03
PTB RRM1/SL UCUUU variants					
PTB RRM1/SL **G**CUUU	0.79	0.9983	0.79	0.9993	0.79 ± 0.01
PTB RRM1/SL UCUU_	0.78	0.9986	0.77	0.9992	0.77 ± 0.01
PTB RRM1/SL UCU**G**U	0.64	0.9937	0.61	0.999	0.63 ± 0.02
PTB RRM1/SL UC**G**UU	0.55	0.9856	0.50	0.9872	0.52 ± 0.04
PTB RRM1/SL UUCG	0.50	0.9795	0.43	0.986	0.47 ± 0.04
PTB RRM1/SLE variants					
PTB RRM1/SL E	0.81	0.996	0.86	0.998	0.84 ± 0.04
PTB RRM1/SL E UUCG	0.56	0.993	0.58	0.996	0.57 ± 0.01

Figure S1. Sequence alignment of PTB and homologues. (A) Alignment between N-terminal region of PTB and its homologues ROD1 and nPTB, alignment performed with CLUSTAL 2.1 online software, http://www.ebi.ac.uk/Tools/msa/clustalw2/. (B) Alignment of complete PTB sequences of a range of vertebrate species showing RRM1 to the start of RRM2. Alignment performed with MAFFT online software, https://mafft.cbrc.jp/alignment/software/ using MAFFT-L-INS-i alignment (v7.372). The PTB sequence used in this study is in bold, secondary structures, green (red for α3). RNP2 and RNP1 motifs located in β1 and β3 are underlined. Residues 76, 85, 88, 151 and 154 are shown in red in the last line summarizing sequence conservation.

A

```
                                                                       β1
PTB      MDGIVPDIAVGTKRGSDELFSTCVTNGPFIMSSNSASAANGNDSKKFKGDSRSAGVPSRVIHIRKLPIDV 70
ROD1     ------------------------------MNSSTPSTANGNDSKKFKRD-RPPCSPSRVLHLRKIPCDV 39
nPTB     MDGIVTEVAVGVKRGSDELLSGSVLSSPNSNMSSMVVTANGNDSKKFKGEDKMDGAPSRVLHIRKLPGEV 70
                                         *.   :********** : :    ****:*:**:* :*

               α1          β2        β3          α2                β4
PTB      TEGEVISLGLPFGKVTNLLMLKGKNQAFIEMNTEEAANTMVNYYTSVTPVLRGQPIYIQFSNHKELKTDS 140
ROD1     TEAEIISLGLPFGKVTNLLMLKGKSQAFLEMASEEAAVTMVNYYTPITPHLRSQPVYIQYSNHRELKTDN 109
nPTB     TETEVIALGLPFGKVTNILMLKGKNQAFLELATEEAAITMVNYYSAVTPHLRNQPIYIQYSNHKELKTDN 140
         ** *:*:**********:******.***:*: :**** ******:.:** **.**:***:***:*****.

                 α3
PTB      SPNQARAQAALQAVNSVQSGNLA- 163
ROD1     LPNQARAQAALQAVSAVQSGSLA- 132
nPTB     TLN-QRAQAVLQAVTAVQTANTPL 163
           *  ****.****.:**:.. .
```

B

```
                                                             β1          α1
sp|P26599|PTBP1 TCVTNGPFIMSSNSAS----AANGNDSKKFKGDSRSAGVPSRVIHIRKLPIDVTEGEVIS Humans
tr|K7AXA8|K7AXA TCVTNGPFIMSSNSAS----AANGNDSKKFKGDSRSAGVPSRVIHIRKLPIDVTEGEVIS Chimpanzee
tr|H9FXJ8|H9FXJ TCVTNGPFIMSS-SNS----AANGNDSKKFKGDSRSAGVPSRVIHIRKLPIDVTEGEVIS Rhesus macaque
sp|Q8WN55|PTBP1 ACVTNGPFIMSGTSAS----TANGNDSKKFKGDSRSAGVPSRVIHIRKLPGDVTEGEVIS Dog
tr|J9P5T5|J9P5T ACVTNGPFIMSSNSAS----AANGNDSKKFKGDNRSAGVPSRVIHIRKLPGDVTEGEVIS Cow
tr|Q3T984|Q3T98 TCVSNGPFIMSS-SAS----AANGNDSKKFKGDNRSAGVPSRVIHVRKLPSDVTEGEVIS Mouse
tr|D3ZB30|D3ZB3 TCVSNGPFIMSS-SAS----AANGNDSKKFKGDNRSTGVPSRVIHVRKLPSDVTEGEVIS Rat
tr|A0A1L1S0D8|A TCVTNGPFIMSSNASS----AANGNDSKKFKGDSRSAGVPSRVIHVRKLPSDVTEAEVIS Chicken
tr|H0YQV3|H0YQV --------------------TANGNDSKKFKGDNRSAGIPSRVIHVRKLPSDVTEAEVIS Zebra Fish
tr|M7AII9|M7AII TCVTNGPFIMSSNSAS----AANGNDSKKFKGDSRSAGVPSRVIHVRKLPSDVTEAEVIS Blind Cave Fish
tr|H3A366|H3A36 -----------SNSAS----AANGNDTKKFKGDNRSSGVPSRVIHVRKLPNDITEAEVIS Western Clawed Frog
tr|Q503D3|Q503D SCISNGPYIMSS---G----AANGNDSKKFKGDIRSPGIPSRVIHVRKLPNDINEAEVIS African Elephant
tr|W5LHY3|W5LHY SCVSNGPYIMSS--------PANGNDSKKFKGDIRSPGIPSRVIHVRKLPSDINEAEVIS Southern Platyfish
tr|M4ANC5|M4ANC SCISNGPYIMNS---------ANGNDSKKFKGDVRSPGVPSRVVHVRKLPNDINEAEVIS Coelacanth
tr|F6YXS8|F6YXS SCVTNGPFIMSNATAGENLYGSNGNDSKKFKGDSRSVAVGSRVIHVRKLPGDVTEAEVIS Zebrafinch
tr|G3UBJ5|G3UBJ SCVPQRPFIMSSSSPS----AANGNDSKKFKVDSRSAGAPSRVVHIRKLPSDVTEGEVIS Sea turtle
                                     :****:**** * ** .  ***:*:**** *:.*.****

                           β2         β3         α2                β4
sp|P26599|PTBP1 LGLPFGKVTNLLMLKGKNQAFIEMNTEEAANTMVNYYTSVTPVLRGQPIYIQFSNHKELK Humans
tr|K7AXA8|K7AXA LGLPFGKVTNLLMLKGKNQAFIEMNTEEAANTMVNYYTSVTPVLRGQPIYIQFSNHKELK Chimpanzee
tr|H9FXJ8|H9FXJ LGLPFGKVTNLLMLKGKNQAFIEMNTEEAANTMVNYYTSVTPVLRGQPIYIQFSNHKELK Rhesus macaque
sp|Q8WN55|PTBP1 LGLPFGKVTNLLMLKGKNQAFIEMHTEEAANTMVNYYTSVTPVLRGQPIYIQFSNHKELK Dog
tr|J9P5T5|J9P5T LGLPFGKVTNLLMLKGKNQAFIEMNTEEAANTMVNYYTSVTPVLRGQPIYIQFSNHKELK Cow
tr|Q3T984|Q3T98 LGLPFGKVTNLLMLKGKNQAFIEMNTEEAANTMVNYYTSVAPVLRGQPIYIQFSNHKELK Mouse
tr|D3ZB30|D3ZB3 LGLPFGKVTNLLMLKGKNQAFIEMSTEEAANTMVNYYTSVAPVLRGQPIYIQFSNHKELK Rat
tr|A0A1L1S0D8|A LGLPFGKVTNLLMLKGKNQAFIEMNTEEAANTMVNYYTTVTPVLRSQPIYIQFSNHKELK Chicken
tr|H0YQV3|H0YQV LGLPFGKVTNLLMLKGKNQAFIEMNTEETANTMVNYYTTVTPVLRSQPIYIQFSNHKELK Zebra Fish
tr|M7AII9|M7AII LGLPFGKVTNLLMLKGKNQAFIEMNTEEAANTMVSYYTTVTPVLRSQPIYIQFSNHKELK Blind Cave Fish
tr|H3A366|H3A36 LGLPFGKVTNLLMLKGKNQAFLEMSTEEAANTMMSYYTTVTPVLRNQAIYVQFSNHRELK Western Clawed Frog
tr|Q503D3|Q503D LGLPFGKVTNLLMLKGKNQAFLEMNTEESAQTMVSYYSSVTPVIRNHPIFMQYSNHKELK African Elephant
tr|W5LHY3|W5LHY LGLPFGKVTNLLMLKGKNQAFLEMNTEEAAQTMVSYYSSVTPVIRNHPIFMQYSNHKELK Southern Platyfish
tr|M4ANC5|M4ANC LGLPFGKVTNLLMLKGKNQAFLELNSEECAQTMVSYYSSVTPVIRNHPIYMQYSTHKELK Coelacanth
tr|F6YXS8|F6YXS LGLPFGKVTNLLMLKGKNQVCMKVYSKKNTFCAFNVCCNINSLLVIERMYWLVYGHARLI Zebrafinch
tr|G3UBJ5|G3UBJ LGLPFGKVTNLLMLKGKNQAFIEMNTEEAANTMVNYYTSVAAVLRGQPIYIQFSNHKELK Sea turtle
                *******************. ::: ::: :   ..   .: .::  . ::     * .*

                           α3                              RRM2 ->
sp|P26599|PTBP1 TDSSPNQARAQAALQAVNSVQSGNLALAASAAAVDAGMAMAGQSPVLRIIVENLFYPVTL Humans
tr|K7AXA8|K7AXA TDSSPNQARAQAALQAVNSVQSGNLALAASAAAVDAGMAMAGQSPVLRIIVENLFYPVTL Chimpanzee
tr|H9FXJ8|H9FXJ TDSSPNQARAQAALQAVNSVQSGNLALAASAAAVDAGMAMAGQSPVLRIIVENLFYPVTL Rhesus macaque
sp|Q8WN55|PTBP1 TDSSPNQARAQAALQAVNSVQSGNLALAASAAAVDAGMAMAGQSPVLRIIVENLFYPVTL Dog
tr|J9P5T5|J9P5T TDSSPNQARAQAALQAVNSVQSGNLALAASAAAVDAGMAMAGQSPVLRIIVENLFYPVTL Cow
tr|Q3T984|Q3T98 TDSSPNQARAQAALQAVNSVQSGNLALAASAAAVDAGMAMAGQSPVLRIIVENLFYPVTL Mouse
tr|D3ZB30|D3ZB3 TDSSPNQARAQAALQAVNSVQSGNLALAASAAAVDAGMAMAGQSPVLRIIVENLFYPVTL Rat
tr|A0A1L1S0D8|A TDNSPNQARAQAALQAVNSVQSGNLALPAPAAAVDAGMAMAGQSPVLRIIVENLFYPVTL Chicken
tr|H0YQV3|H0YQV TDNSPNQARAQAALQAVTQVQAGAVALAATAAAVDAGMAMAGQSPVLRIIVENLFYPVTL Zebra Fish
tr|M7AII9|M7AII TDNSPNQARAQAALQAVNSVQSGNLALSASAAAVDAGMAMAGQSPVLRIIVENLFYPVTL Blind Cave Fish
tr|H3A366|H3A36 TDNSPNQVRAQAALQAVNAVQSANVVLTTASAAIDPGVGMSGQSPVLRIIVENLFYPVTL Western Clawed Frog
tr|Q503D3|Q503D TDNSPNQVRAQAALQAVNAVQTGGMSL----AAID-GAGMGSQSPVLRVIVENLFYPVTL African Elephant
tr|W5LHY3|W5LHY TDNSPNQVRAQAALQAVNAVQTGSMAI----AGVD-PSGVTGPSPVLRVIVENLFYPVTL Southern Platyfish
tr|M4ANC5|M4ANC TDNSPNQVRAQAALQAVNALHGGGMGSMAIPTDASSLAGATAQSPVLRVIVENLFYPVTL Coelacanth
tr|F6YXS8|F6YXS LNKAAKKMKAQAALQAVNSVVSGTTALSASAAAVDAGMAMSGQSPVLRIIVENLFYPVTL Zebrafinch
tr|G3UBJ5|G3UBJ TDSSPNQARAQAALQAVNSVQSGSLALAASAAAVDAGMAMAGQSPVLRIIVENLFYPVTL Sea turtle
                 :.:.:: :********. :  .        :  .   .  .*****:***********
```

Figure S2: Isothermal calorimetry titration profile of PTB RRM1 in complex with SL UCUUU fitted with a two binding site model.

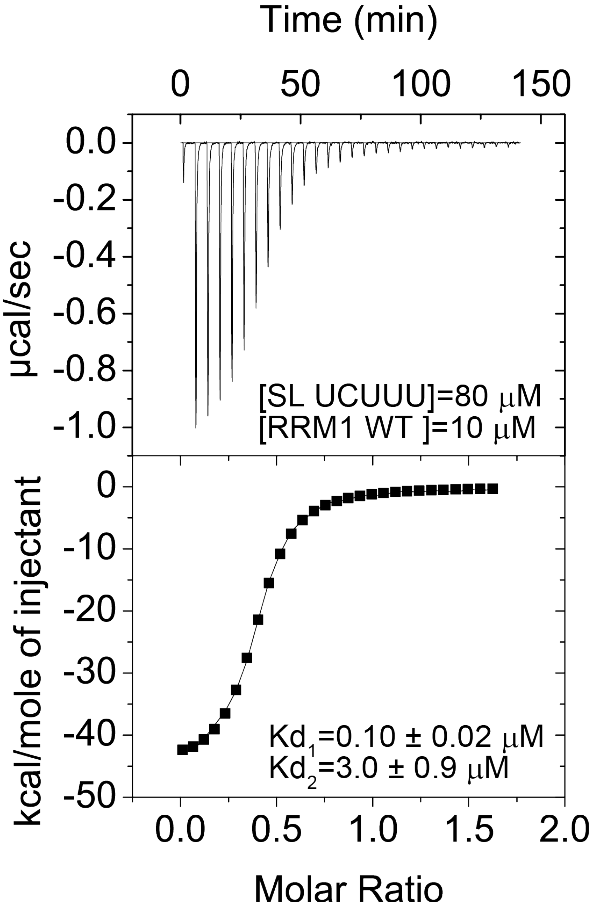

Figure S3. ^{1}H-^{15}N HSQC spectrum overlay of PTB RRM1 in complex with SL UCUUU at equimolar ratio (red) and a protein:RNA ratio of 2:1 (yellow).

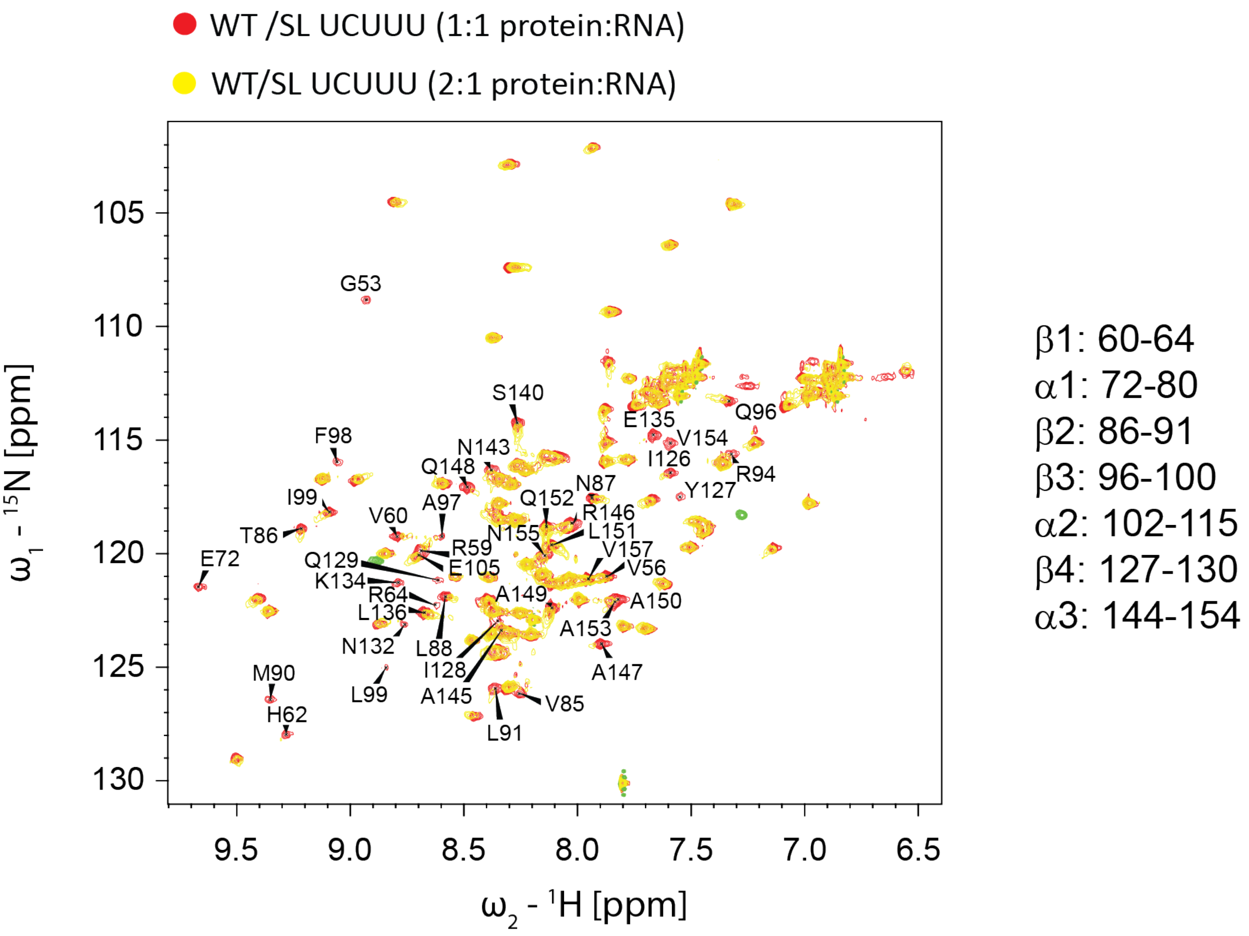

Figure S4. (A) ^{1}H-^{13}C HSQC spectrum of SL UCUUU specifically ^{13}C labeled at sugar position U10, U12 and U14 in complex with PTB RRM1. (B) ^{1}H-^{13}C HSQC spectrum of the aromatic region of fully $^{13}C^{15}N$ labeled SL UCUUU in complex with PTB RRM1. (C) From left to right, 2D plane of the 3D ^{13}C f1-edited,f3-filtered NOESY of SL UCUUU specifically ^{13}C labeled at the sugar positions U10, U12 and U14 in complex with PTB RRM1 and 2D plane of the 3D 13C f1-edited, f3-filtered NOESY of $^{13}C^{15}N$ labeled PTB RRM1 in complex with SL UCUUU. (D) 2D plane of a 3D ^{13}C edited NOESY with 3-9-19 WATERGATE water suppression to detect NOEs between ^{1}H attached to ^{13}C of the protein and RNA iminos acquired at 5°C.

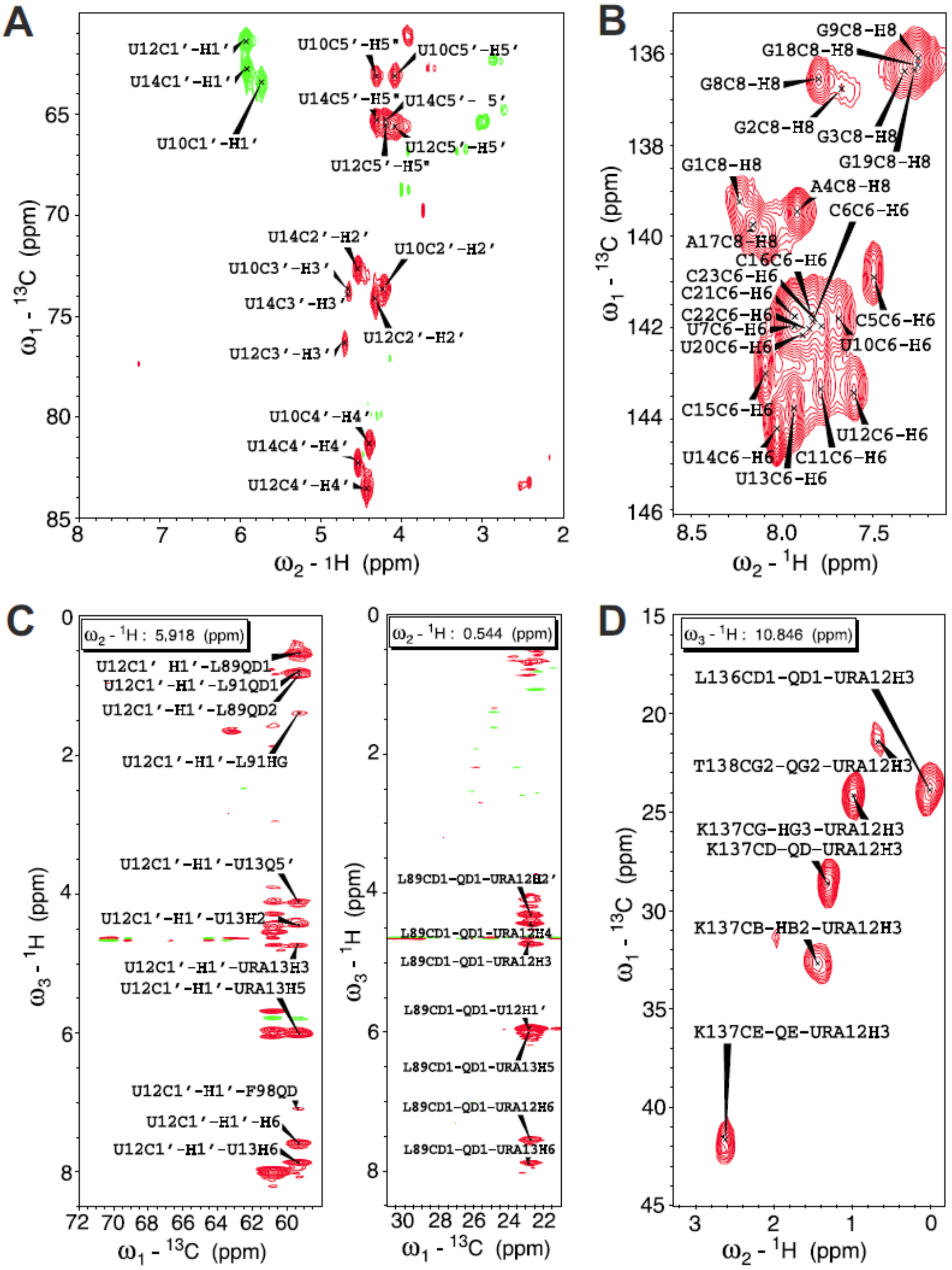

Figure S5. (A) Network of intermolecular NOE restraints for C11, U12 and U13. (B) 2D through-bond spectrum, which correlates uracil RNA iminos (H3) to H5 resonances (left), aligned with the C5-H5 region of a ^{13}C-^{1}H HSQC of $^{13}C^{15}N$ labeled SL UCUUU RNA acquired at 5°C (right). (C) Overlay of H5-H6 region of 2D TOCSY spectra from SL UCUUU in the free and protein bound states at 40°C.

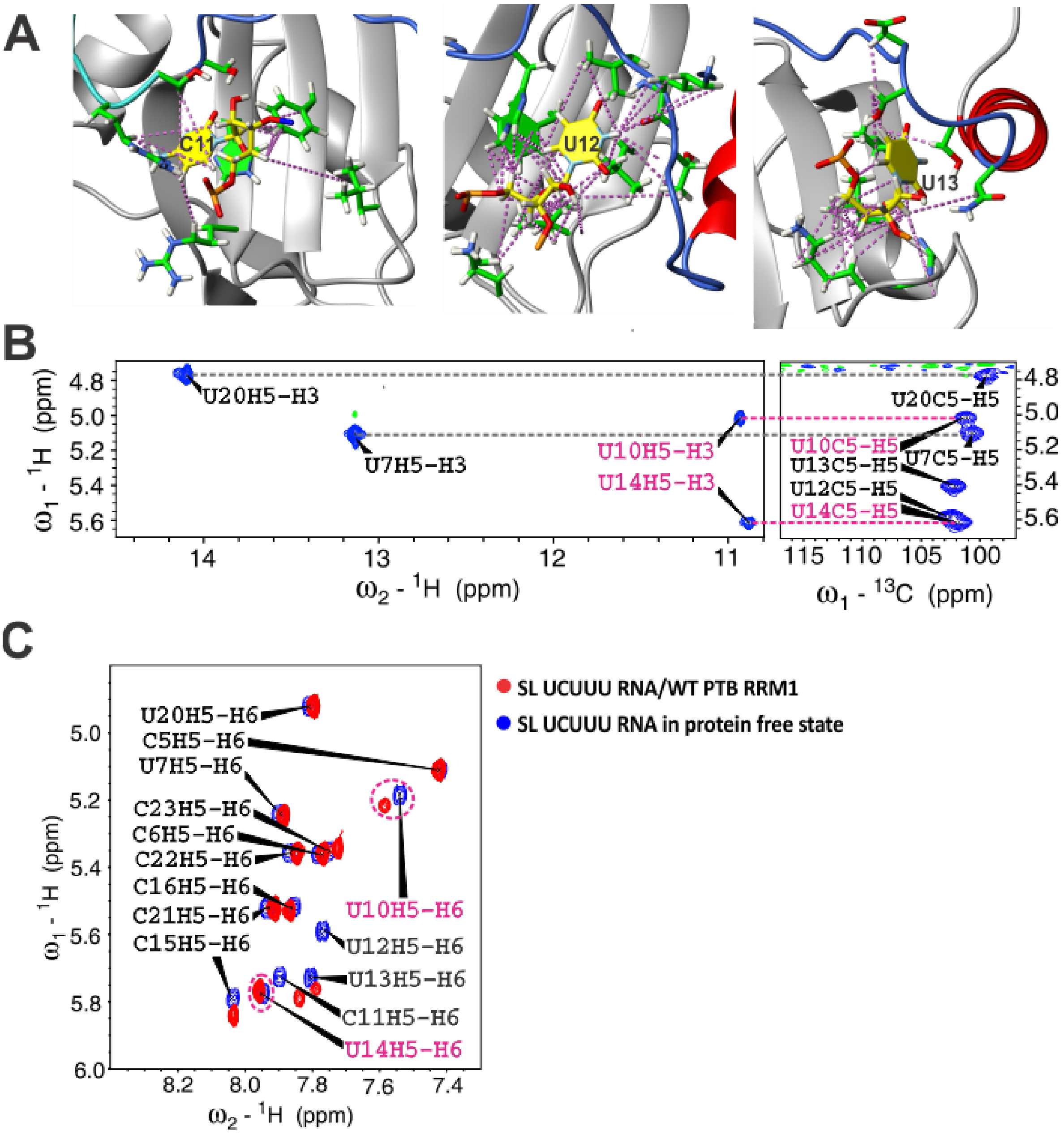

Figure S6. (A) Isothermal titration calorimetry profiles for the RNA and protein mutants performed at 30°C. (B) Linear correlation between the entropic and enthalpic terms determined by ITC methods from wild type and mutant complexes.

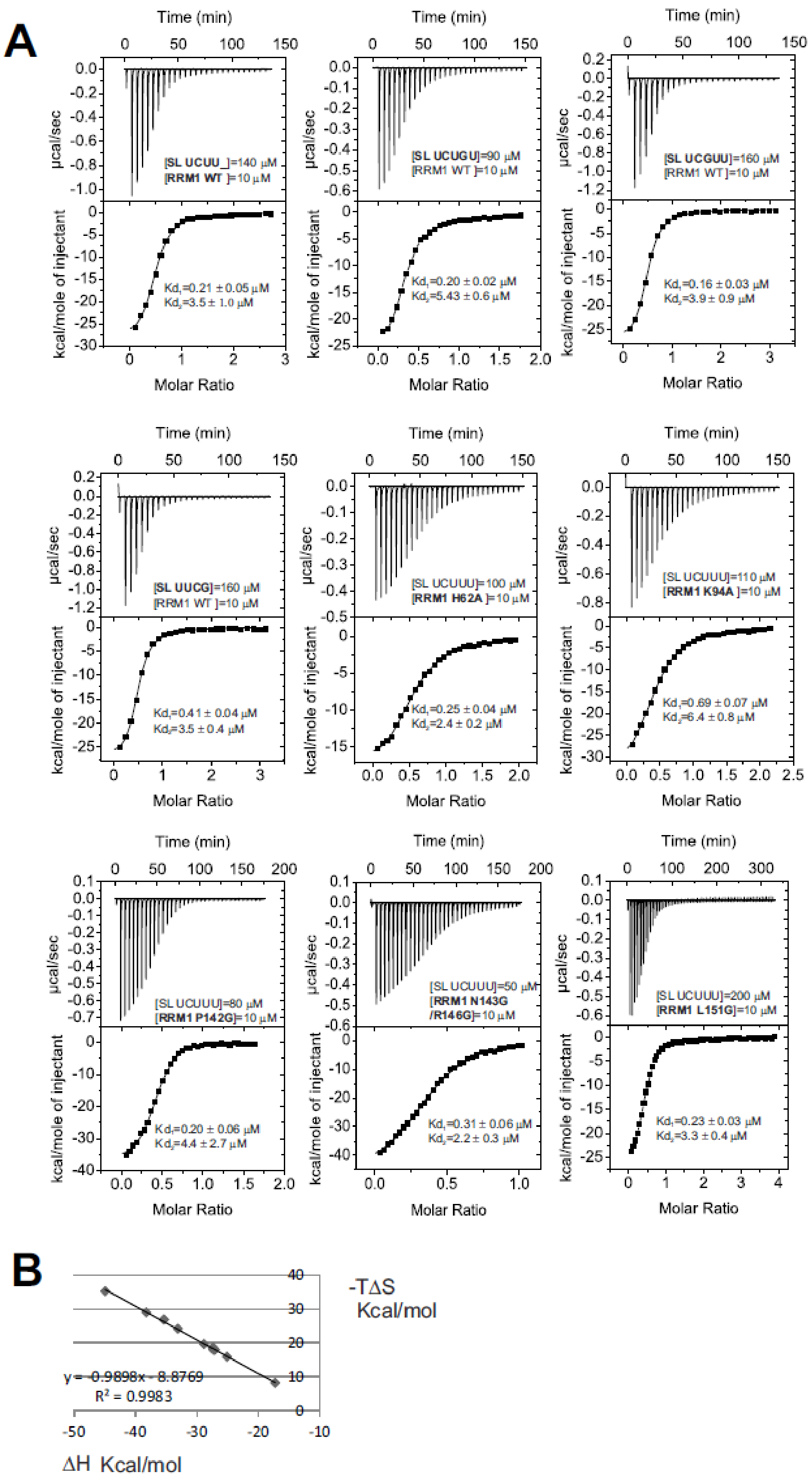

Figure S7. Superposition of ^1H-^{15}N HSQC spectra of WT PTB RRM1 protein in the free and SL UCUUU bound states and the mutant proteins designed to impact α3 helix formation in the free state (NMR data acquired at 40°C). Amide chemical shift changes of Glu72, Val85 and Val154 are highlighted.

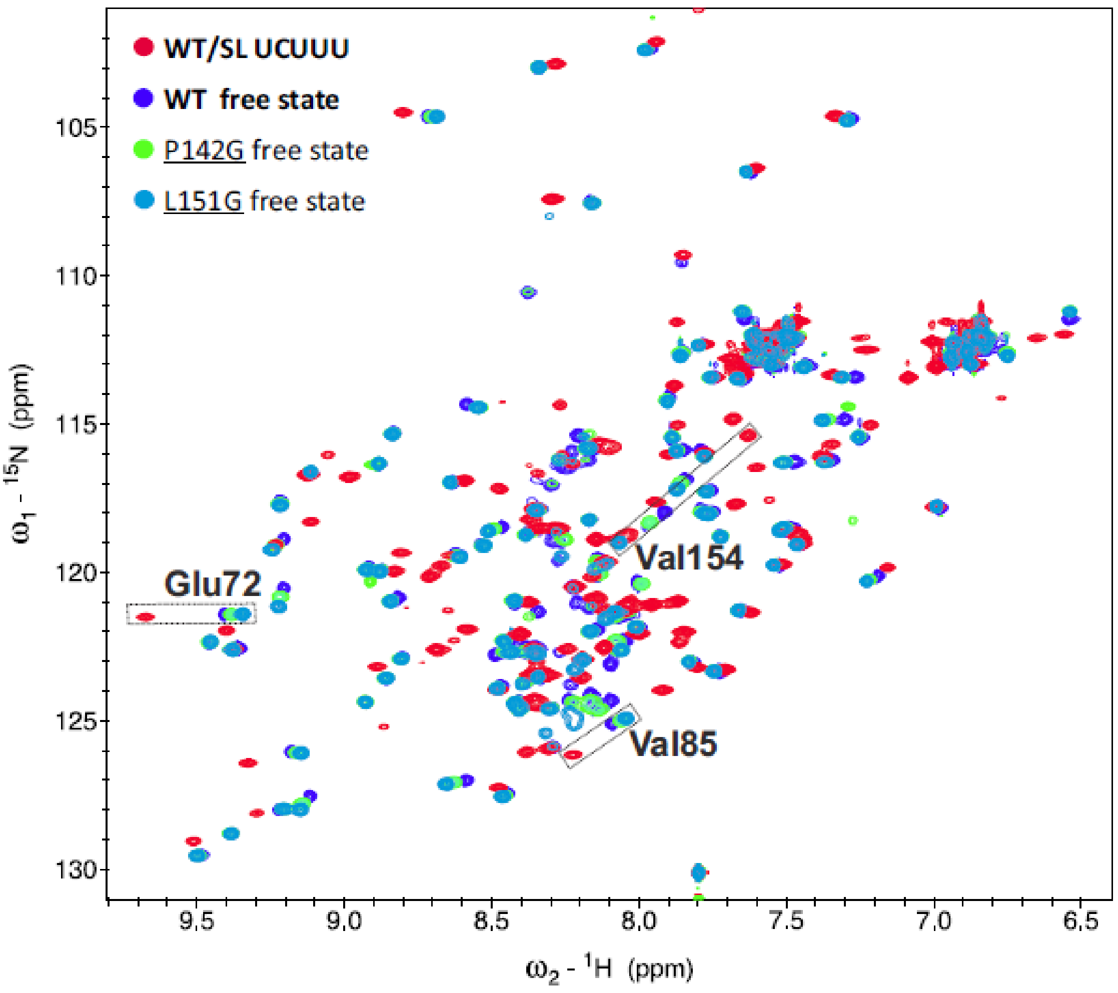

Figure S8. Overview of co-linear chemical shift perturbation behavior observed upon protein and RNA mutations. (A) Superposition of ^{1}H-^{15}N HSQC spectra of WT PTB RRM1 protein and the L151G mutant in the free and SL UCUUU bound states and P142G mutant in SL UCUUU bound state (NMR data acquired at 40°C). Amide chemical shift changes of Glu72, Val85 and Val154 are highlighted. For the L151G mutant, Glu72 and Val85 show slight shifts due to SL UCUUU binding which are opposed to the direction induced by RNA binding in the other proteins. (B) Superposition of ^{1}H-^{15}N HSQC spectra of WT PTB RRM1 protein in the free and SL UCUUU bound states and the RNA mutant complexes (NMR data acquired at 40°C). Amide chemical shift changes of several residues from α helices and β strands are highlighted.

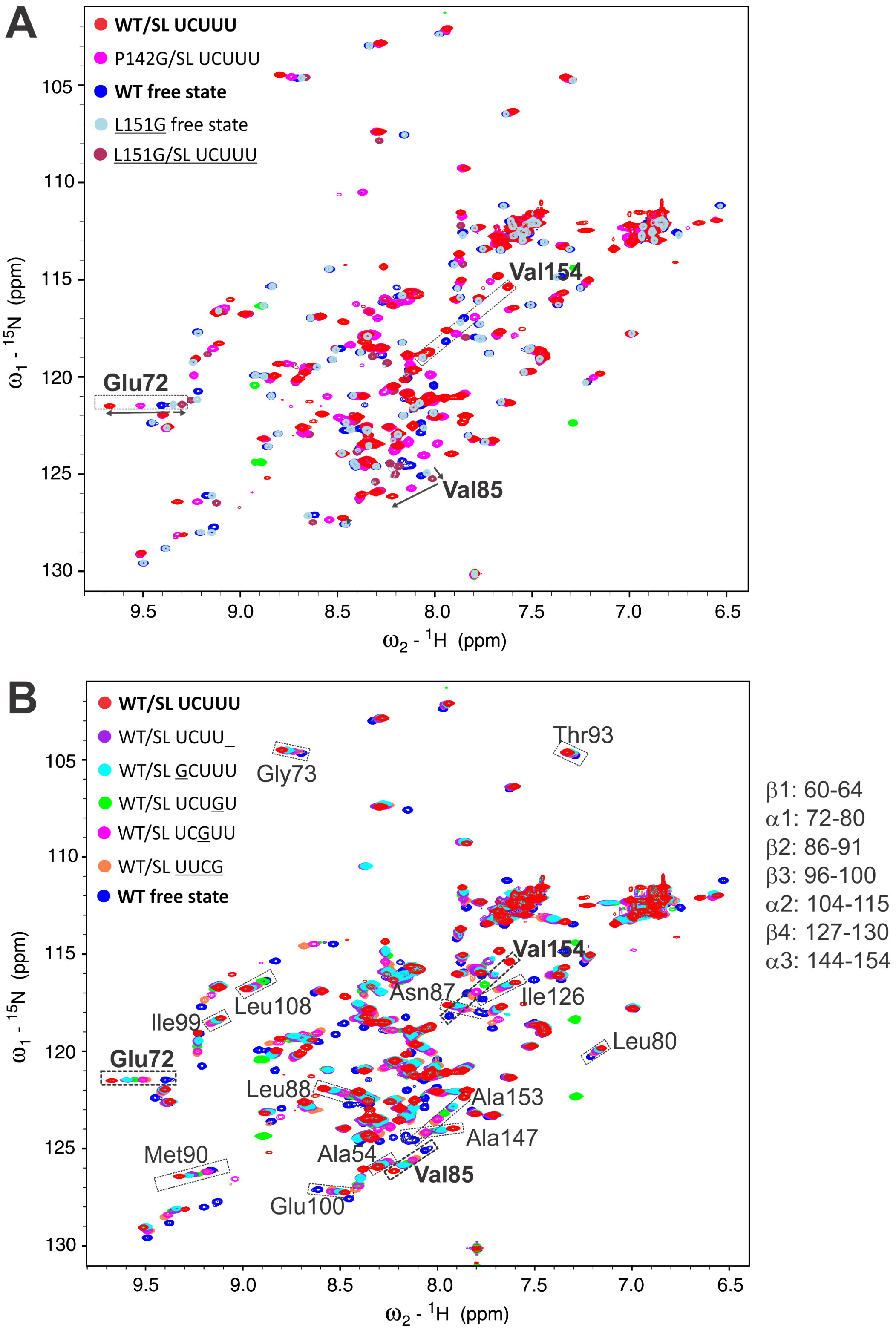

Figure S9.(A) Overlay of the amide chemical shift perturbation mappings of the wild type and two mutant PTB RRM1 proteins in complex with SL UCUUU demonstrating the absence of significant chemical shift changes in the α3 region of the L151G mutant, and the docking region of α3 on RRM1 (residues 72-84). (B) Overlay of the combined amide chemical shift perturbations of the wild type PTB RRM1 protein in complex with WT and mutant RNA hairpins showing the progression of increasing shift changes in the α3 region from SL UUCG with the smallest shifts to SL UCUUU with the largest shifts. (C) Superposition of the H5-H6 region of 2D TOCSY spectra of SL UUCG in the free and protein-bound states acquired at 40 °C and secondary structure of SL UUCG mutant.

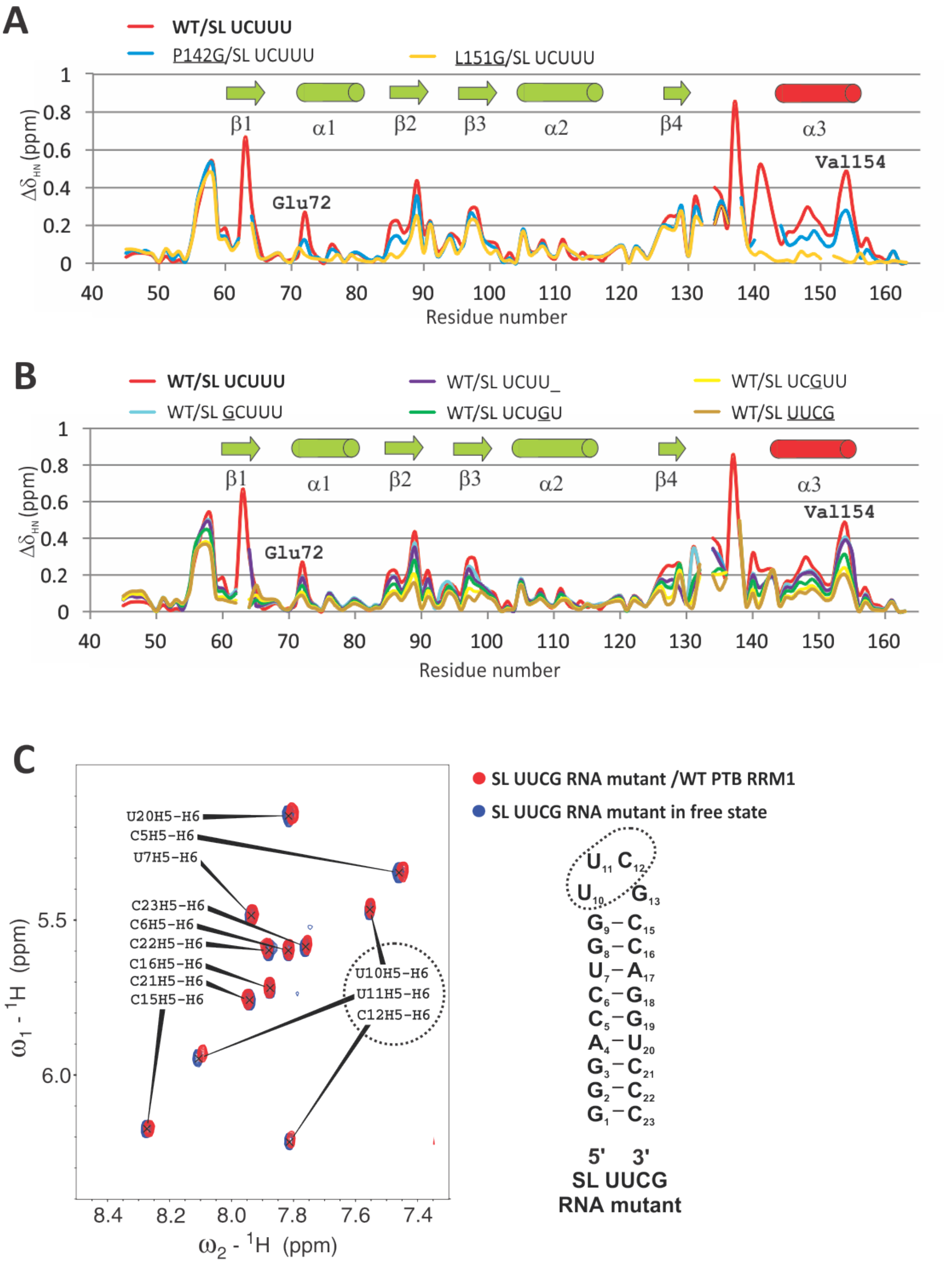

Figure S10. The amide chemical shift difference between WT PTB RRM1/SL UCUUU complex and L151G mutant for residues 72-86 (^{15}N of α1-β2 segment) (A), and residues 147-154 (^{1}H and ^{15}N of α3 helix) (B,C) plotted versus shift difference between WT PTB RRM1, or its complexes with SL RNA mutants, and L151G. The color code is the same as that employed to represent different samples in the ^{1}H-^{15}N HSQC spectra in Figure 6E. Fitting parameters for the linear least squares fitting in Fig. 6E and *A* are given in Tables S2, and for *B* & *C* in Table S3. In *C*, data points for residues 150-152 were omitted due to the local effect of the L151G mutation on ^{15}N shifts of these residues.

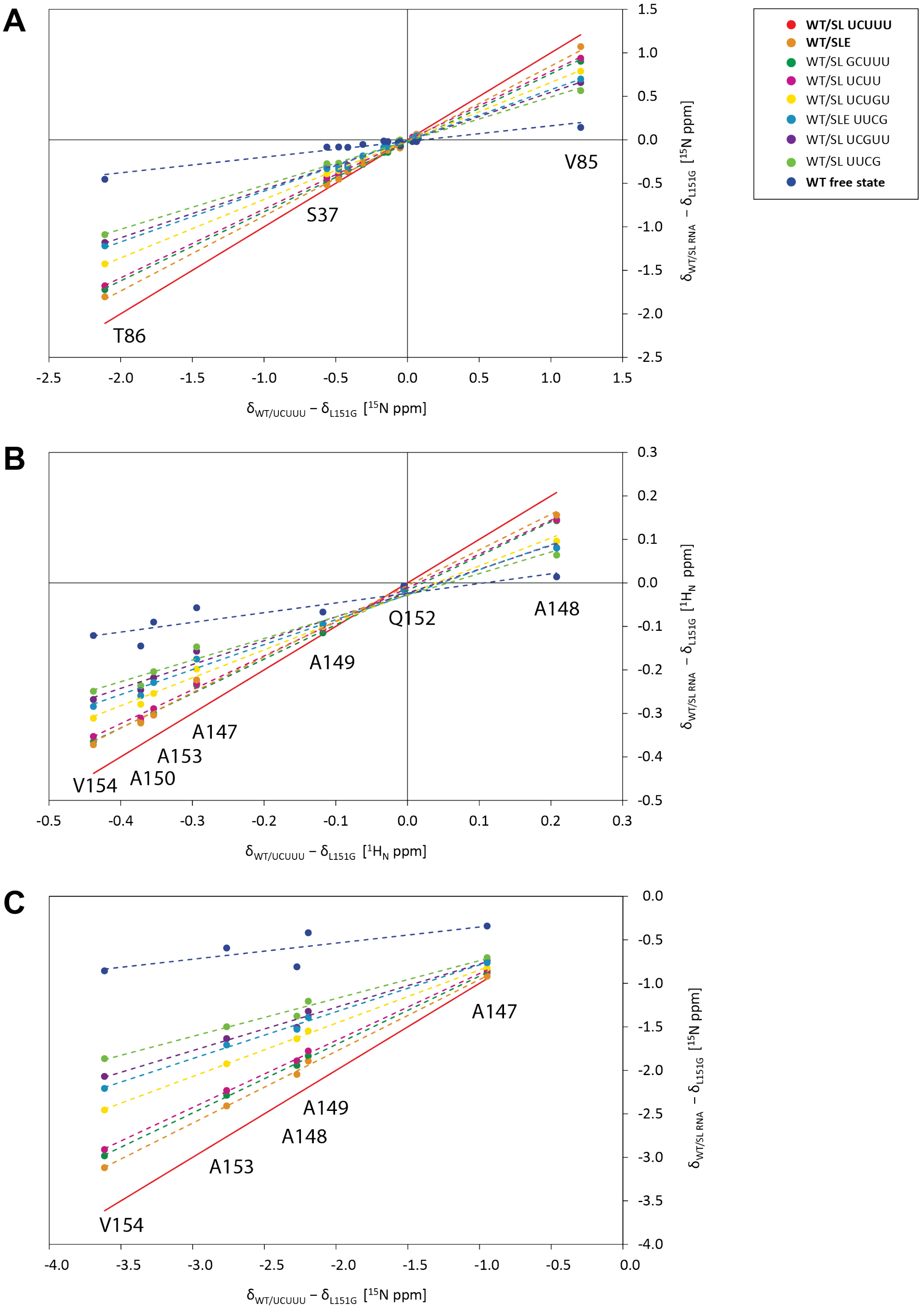

Figure S11. (A) Amide chemical shift perturbations of WT PTB RRM1 in complex with SL UCUUU (spectra acquired at 40°C) and in complex with CUCUCU RNA (spectra acquired at 45°). (B) Smoothed secondary chemical shift trends ($^{13}\Delta C\alpha - ^{13}\Delta C\beta$) versus the protein sequence of the L151G mutant in free state, and WT PTB RRM1 in the free state, in complex with SL UCUUU and CUCUCU RNA. NMR data were acquired at 25°C for L151G, 40°C for WT protein in free state and in complex with SL UCUUU and at 45°C for WT protein in complex with CUCUCU RNA.

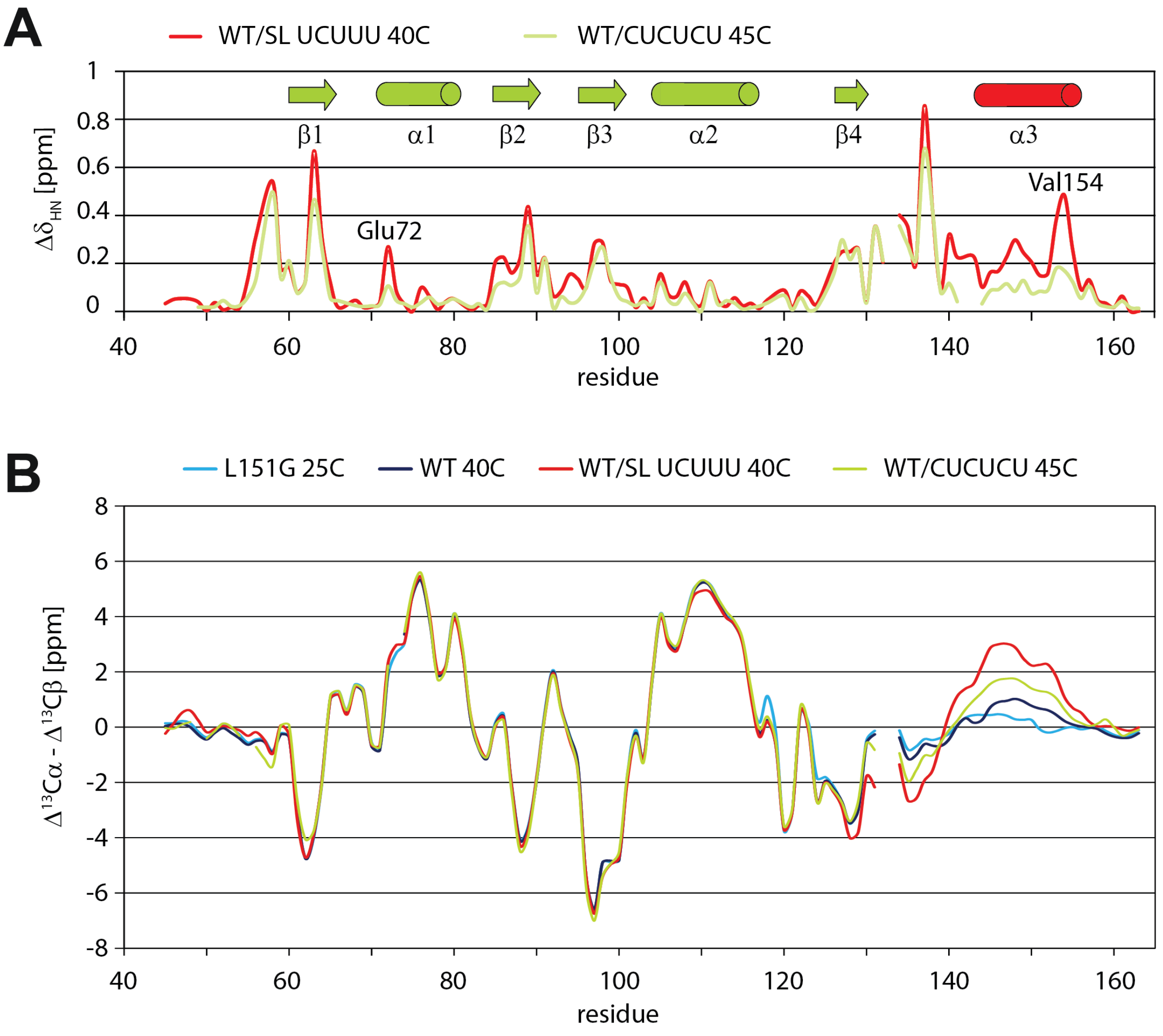

Figure S12. (A) Superposition of ^{1}H-^{15}N HSQC spectra of PTB RRM1 in complex with SL UCUUU (red) and hsa-mir-136 microRNA (yellow). Closely similar positions of Glu72 in α1 and Val154 in α3 indicates that hsa-mir-136 microRNA is able to induce nearly the same α3 helix content as UCUUU SL RNA. NMR data were acquired at 40°C. (B) Stemloop secondary structure of hsa-mir miRNA , and (C) SL UCUUU. The pyrimidine tracts in the apical loops are highlighted in magenta.

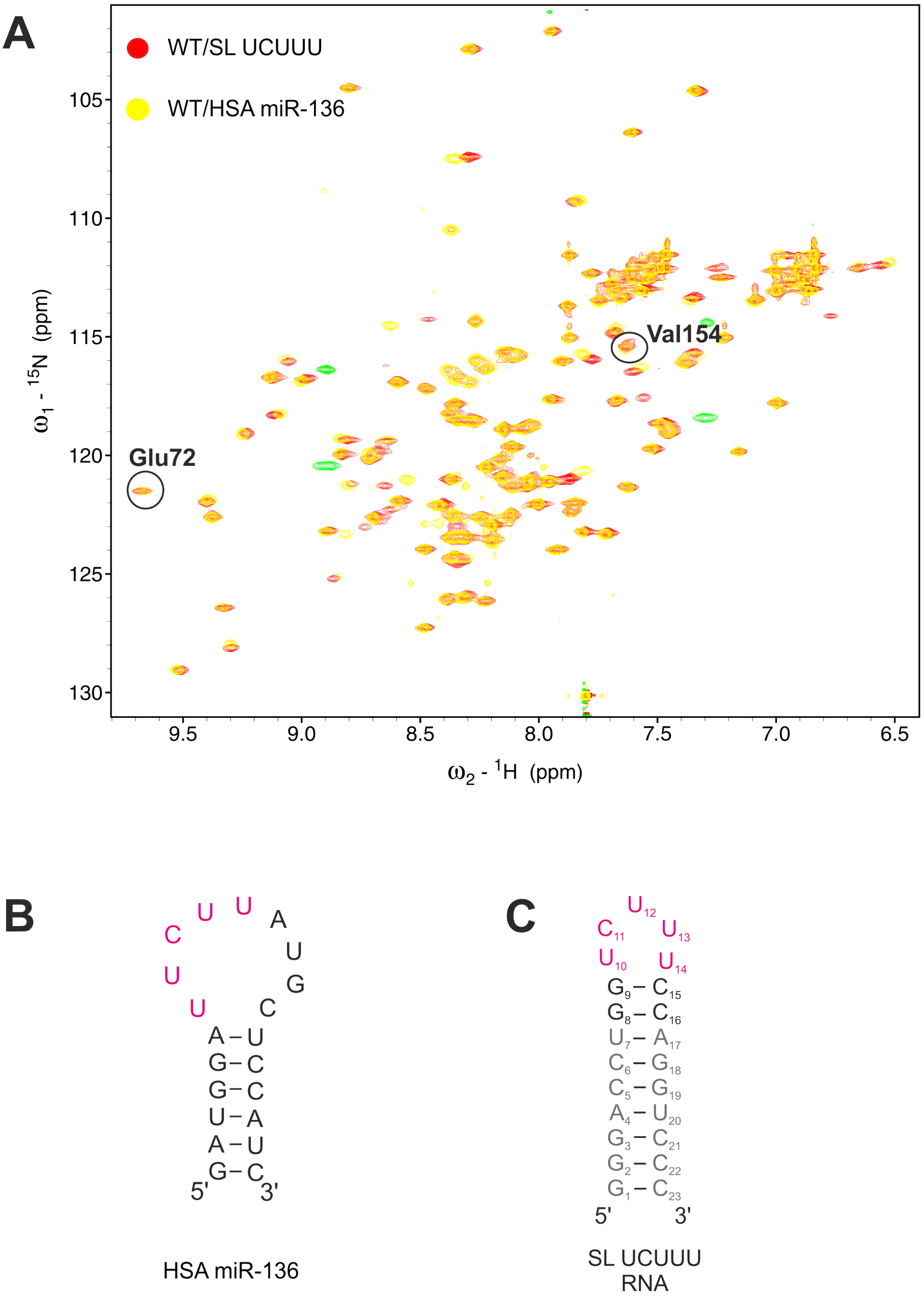

Figure S13. (A) Superposition using backbone coordinates in base-paired regions of the structures of SL UCUUU in the PTBRRM1 bound state (yellow) and SL UUCG in its free state (blue, RCSB accession code: 1HLX). (B) The same superposition showing only the structure of PTBRRM1 in the SL UCUUU-bound state and the structure of SL UUCG, illustrating the complementarity of the contacts between the protein and the RNA backbone. RNA atoms are color coded by type: carbon, yellow; oxygen, red; nitrogen, blue; phosphorus, orange. Selected protein side chains are shown with the color code: carbon, green; oxygen, gold, nitrogen, blue.

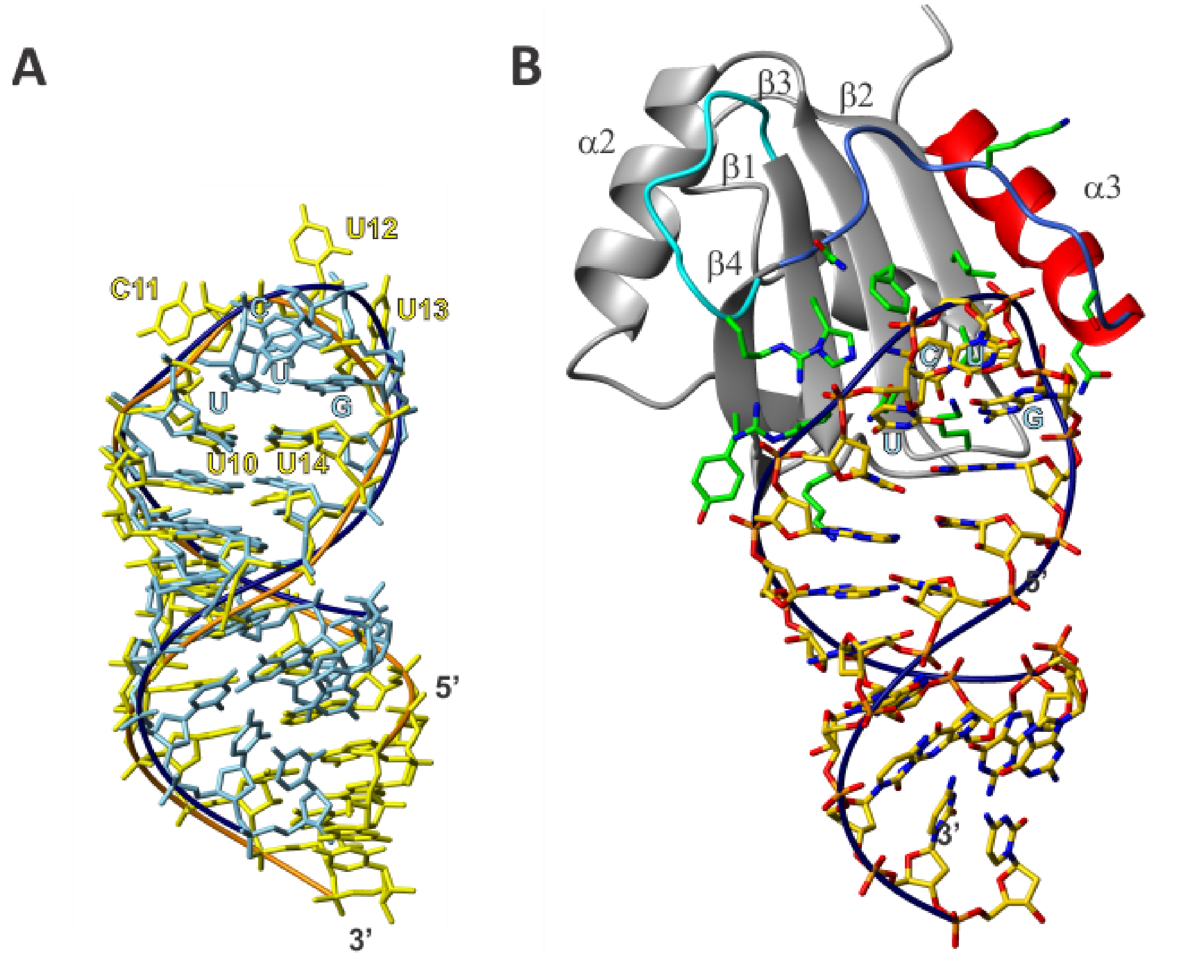

Supplement: gkaa155_Supplemental_File [file gkaa155_supplemental_file.pdf]
